# An Extracellular Subtilase Switch for Immune Priming in Arabidopsis

**DOI:** 10.1371/journal.ppat.1003445

**Published:** 2013-06-20

**Authors:** Vicente Ramírez, Ana López, Brigitte Mauch-Mani, Ma José Gil, Pablo Vera

**Affiliations:** 1 Instituto de Biología Molecular y Celular de Plantas, Universidad Politécnica de Valencia-C.S.I.C, Ciudad Politécnica de la Innovación, Valencia, Spain; 2 University of Neuchâtel, Faculty of Sciences, Institute of Botany, Neuchâtel, Switzerland; Purdue University, United States of America

## Abstract

In higher eukaryotes, induced resistance associates with acquisition of a priming state of the cells for a more effective activation of innate immunity; however, the nature of the components for mounting this type of immunological memory is not well known. We identified an extracellular subtilase from Arabidopsis, *SBT3.3*, the overexpression of which enhances innate immune responses while the loss of function compromises them. *SBT3.3* expression initiates a durable autoinduction mechanism that promotes chromatin remodeling and activates a salicylic acid(SA)-dependent mechanism of priming of defense genes for amplified response. Moreover, *SBT3.3* expression-sensitized plants for enhanced expression of the *OXI1* kinase gene and activation of MAP kinases following pathogen attack, providing additional clues for the regulation of immune priming by SBT3.3. Conversely, in *sbt3.3* mutant plants pathogen-mediated induction of SA-related defense gene expression is drastically reduced and activation of MAP kinases inhibited. Moreover, chromatin remodeling of defense-related genes normally associated with activation of an immune priming response appear inhibited in *sbt3.3* plants, further indicating the importance of the extracellular SBT3.3 subtilase in the establishment of immune priming. Our results also point to an epigenetic control in the regulation of plant immunity, since *SBT3.3* is up-regulated and priming activated when epigenetic control is impeded. SBT3.3 represents a new regulator of primed immunity.

## Author Summary

Following a first encounter with a pathogen, higher eukaryotes develop enhanced resistance to subsequent infections by a broad spectrum of pathogens. This type of induced resistance (IR) exhibits memory characteristics after the first encounter with a pathogen —training effect— and appears evolutionarily conserved. IR response components must reside in a plant's capacity to reprogram gene expression. Among the mechanisms involved in immune-related transcriptional reprogramming, the importance of chromatin remodelling is emerging. Recent studies indicated a causal link between priming and chromatin remodelling, pointing to a histone memory for information storage in the plant immune response. These results emphasized the importance of epigenetic control as an additional layer of complexity in plant immunity. However, the nature of the components for activating this type of immunological memory remains elusive. Here, in a search aiming to identify cellular factors integral in regulating immunity in Arabidopsis, we found that the *SBT3.3* gene, encoding an extracellular subtilase enzyme, is pivotal for establishing plant immune priming. Moreover, based on molecular and genetic evidences, our results indicate that SBT3.3 expression is under epigenetic control thus highlighting the importance of this mechanism of gene regulation in the control of plant immunity and IR.

## Introduction

Plants are continuously faced with threats from pathogenic microorganisms. They counteract microbial infections via activation of an innate immune system in a timely, accurate, and effective manner following pathogen recognition. The innate immune response is thought to act naïvely to individual pathogen encounters and is dependent on the recognition of broadly conserved molecular features, known as microbe-associated molecular patterns (MAMPs), by plasma membrane proteins known as pattern recognition receptors (PRRs). PRR perception of MAMPs at the cell surface leads to a pattern-triggered immune response called PTI [1]. PTI is characterized by the rapid generation of ion fluxes, production of reactive oxygen species (ROS), phosphorylation cascades, and a transcriptional reprograming that favors defense responses over routine cellular requirements [2]. The defense programme is ultimately controlled through the build-up of specific signalling hormone blends, of which salicylic acid (SA) and jasmonic acid (JA) are particularly important, and eventually establish a broad systemic alert state throughout the plant.

Plants develop heightened activation of the innate immune response state resulting from the initial infection manifested in the form of enhanced resistance to subsequent infections by a broad spectrum of pathogens. This type of induced resistance (IR) or cross-protection exhibits memory characteristics after the first encounter with a pathogen - training effect - and appears evolutionarily conserved, even outside the plant kingdom. Netea *et al.* [3] coined the term “trained immunity” to differentiate it from “innate immunity” (as it is induced only secondarily in hosts that have previously encountered a primary infection), or from “adaptive immunity” (as this implies specificity through T and B cells). In plants, two distinct types of this resistance form have been described: systemic acquired resistance (SAR), and induced systemic resistance (ISR) [4], [5]; both represent a functional immune acclimation requiring the defense response regulator NPR1.

Particularly relevant in IR responses is the observation that defence genes, in both the local (infected) and distal tissue, respond to much lower levels of a pathogenic stimulus in a more rapid and robust manner than controls, thus revealing a “priming” phenomenon. In fact, priming has long been known as a component of IR responses in plants [6], [7] and mammals [8]–[10], and more recently in invertebrates, which like plants lack adaptive immunity [11]. Similarly, Arabidopsis mutants attenuated in pathogen defense (i.e. *npr1*) are also compromised in priming [12], [13]. Organic and inorganic compounds can also induce this form of resistance in plants [14]. Among these, azelaic acid [15], SA, and its functional analogue benzo(1,2,3)thiadiazole-7-carbothioic acid S-methyl ester (BTH) [16], or the non-protein amino acid β-aminobutyric acid (BABA) [17] have attracted marked interest as they potentiate pathogen-specific defense mechanisms, and induction of a primed state. However, very little is known about the molecular mechanism(s) and signals that set a priming state in motion, or the identity of molecular components that pertain to the maintenance of a long lasting immune primed state, such as SAR.

Conrath *et al.* [18] hypothesized that IR or cell priming could be built on the accumulation of dormant or inactive signalling proteins, integral in signal amplification that becomes operative following a challenge with another pathogen, thereby initiating signal amplification leading to a faster and stronger activation of defense responses. However, the identity of such signalling components remains elusive. Interestingly, Beckers *et al.* [12] have shown that during development of BTH induced resistance in Arabidopsis, priming is associated with accumulation of inactive proteins of mitogen-activated protein kinases (MPKs), MPK3 and MPK6. Exposure to the challenges of biotic and abiotic stressors results in stronger activation of the two kinases in primed plants relative to non-primed plants, which is linked to enhanced defense gene expression. Priming of defense gene expression was reduced in *mpk3* or *mpk6* mutants, showing that pre-stress deposition of a MPK cascade is a critical step in priming plants for a full defense response induction during IR [12].

Essential IR response components must rely in a plant's capacity to reprogram gene expression. Among the mechanisms involved in immune-related transcriptional reprogramming, the importance of chromatin remodeling and covalent histone modifications is emerging [19]. Jaskiewicz *et al.* [20], reported that during primed BTH immunity, increased acetylation of histone H3 at Lys-9 (H3K9ac) and trimethylation of histone H3 at Lys-4 (H3K4me3) was detected at promoter regions of several SA-responsive genes encoding transcription factors (i.e. WRKY6, WRKY29, and WRKY52). Similarly, constitutively increased H3K4me3 and H3K9ac mark setting in chromatin of the SA-dependent *PR1* gene was initially reported in *sni1* (*suppressor of nrp1-1, inducible 1*) mutant [21]. The settling of these histone modifications lead chromatin into a suitable state for efficient SA-responsive gene induction when needed. The results also indicated a causal link between priming and chromatin remodeling, pointing to a histone memory for information storage in the plant stress responses [19]. On the other hand RNA Polymerase V is an enzyme critical in the epigenetic RNA-directed DNA methylation (RdDM) pathway and is involved in regulating both DNA methylation and histone modifications [22]. In this context, López *et al.* [23] reported that RNA Polymerase V defective mutants carry a constitutive priming phenotype where SA-related defense genes are poised for enhanced activation via similar H3K4me3 and H3K9ac histone modifications in their promoters. These results emphasized the importance of epigenetic control as an additional layer of complexity in plant immunity and IR regulation [23]. Furthermore, DNA methylation has been implicated in the transmission of a priming state or stress memory, endowing progeny of pathogen-inoculated plants with heightened resistance (transgenerational IR), suggesting plants can inherit priming sensitization [24], [25].

In the present study, we report on identification and characterization of the inducible Arabidopsis subtilase SBT3.3 to characterize additional cellular components mediating initiation and or/maintenance of primed immunity. This extracellular proteolytic enzyme serves a signaling role in establishing immune priming. The mechanism subsequently activates chromatin remodeling and defense genes become poised for enhanced activation following pathogen attack. Our study provides strong evidence that SBT3.3 is a primary switch in immune priming, and it may represent one of the missing components in systemic IR establishment.

## Results

### The Arabidopsis subtilase gene *SBT3.3* is up-regulated in the *csb3* mutant

The Arabidopsis enhanced disease resistance *csb3* (*constitutive subtilisin3*) mutant [26] was isolated during a search for negative disease resistance regulators in a mutant screening that evaluated constitutive expression of GUS activity driven by the 5′ promoter region of a pathogen-induced subtilase gene (*P69C*) from tomato plants [27]. Arabidopsis possess fifty-six highly similar genes encoding subtilases [28], therefore constitutive expression of the Arabidopsis gene homologous to *P69C* would be similarly up-regulated in the *csb3* mutant. Constitutively expressed genes differentially expressed in the *csb3* mutant with respect to wild-type plants were identified by microarray analysis of RNA transcripts using ATH1 Affymetrix chips. The microarray analysis (NCBI GEO Series number GSE35507) identified one hundred down-regulated genes and 367 up-regulated genes in the *csb3* mutant (Supplemental Table S1 and Figure S1). Among the genes up-regulated ≥2-fold (p values<0.05) in the *csb3* mutant, we identified 23 genes that could be linked to disease resistance and SA-mediated responses based on published results (Supplemental Table S2). It was notable that among them one encoded a subtilase: *SBT3.3* (At1g32960). Moreover, public microarray data mining showed that *SBT3.3*, out of the 56 paralogous subtilases from Arabidopsis, with the exception of At1g32940, is the one showing strongest response to pathogen attack and to pathogen-related stress signals (Supplementary Figure S2).

**Figure 1:**
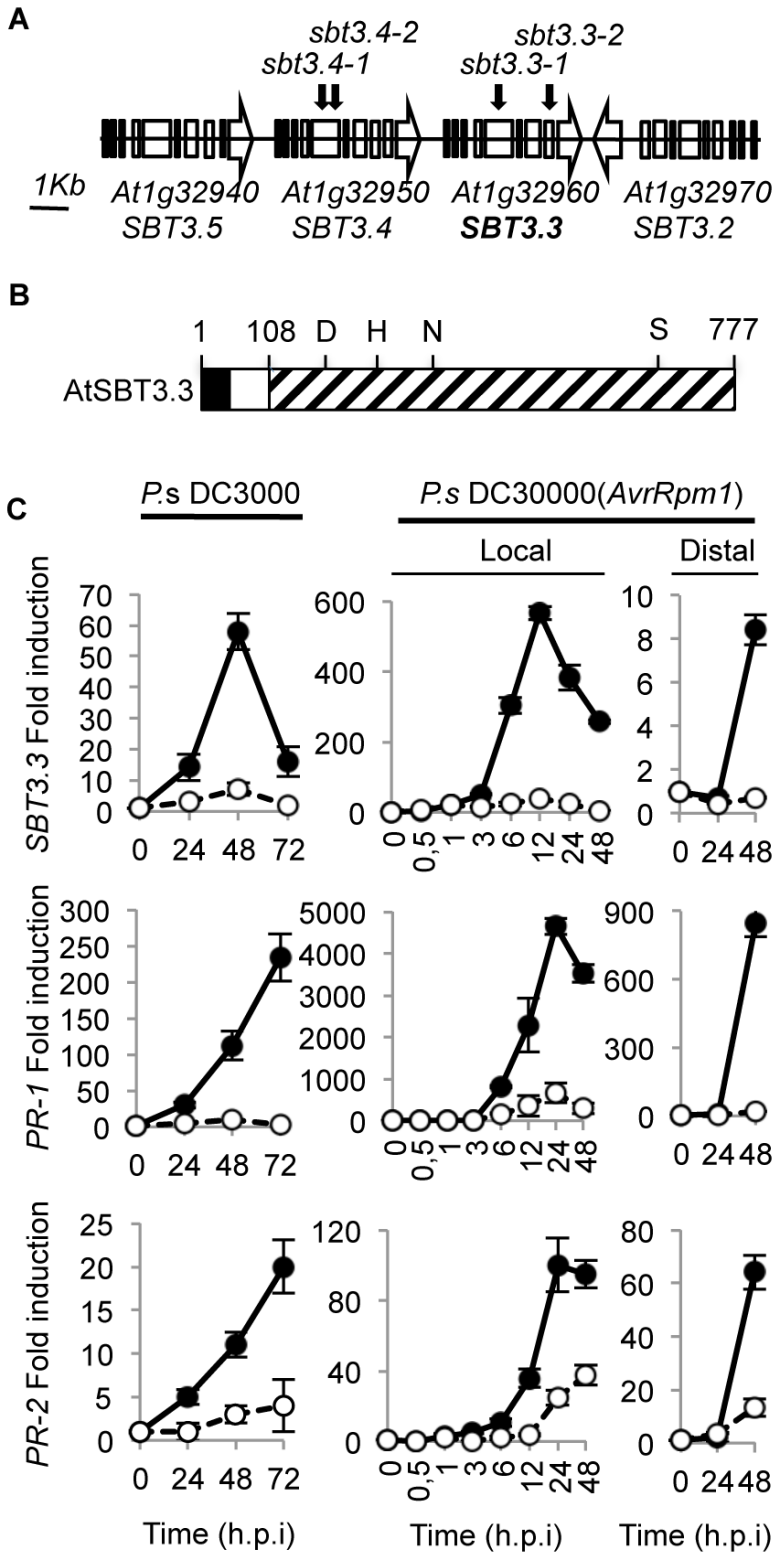
*SBT3.3* genome organization and induced expression following *P.* **syringae** **DC3000 infection.** **A**) Four *SBT3.3*-like open reading frames sequences (named as *SBT3.5, SBT3.4, SBT3.3 and SBT3*) are arranged in tandem in chromosome I. The distances are only approximate. Arrowheads indicate direction of transcription. Black arrows above the genes show position of T-DNA insertions rendering the *sbt3.4* and *sbt3.3* mutant alleles. (**B**) Schematic representation of the SBT3.3 preproenzyme structure. Areas marked respectively in black, white and stippled indicate the signal peptide, propeptide, and mature peptide regions. Numbers depict positions of amino acid residues from the N-terminus. The amino acids forming the catalytic triad (D, H, and S) and the conserved N residues are marked. (**C**) RT-qPCR analyses showing local induction of *SBT3.3*, *PR-1*, and *PR-2* gene expression upon infection with virulent *Ps*DC3000, and both local and distal induction following infection with the avirulent *Ps*DC3000 (*AvrRpm1*) strain. Filled circles represent inoculated plants, and empty circles represent mock-inoculated plants (controls). Data represent mean ± SD, n = 3 replicates. Expression was normalized to the constitutive *ACT2* gene, then to expression at time 0 in Col-0 plants.

Coincident with what has been described in the tomato genome, where the *P69C* subtilase clusters with three additional P69C-like ORFs (i.e. *P69A*, *P69D*, *P69C*, and *P69B*) [29], the *SBT3.3* subtilase gene was similarly embedded in a genomic cluster encompassing three additional subtilases (i.e. *SBT3.5*, *SBT3.4*, *SBT3.3* and *SBT3.2*) in Chromosome 1 (Fig. 1A). Thus, it seems very likely that the Arabidopsis SBT3.3 subtilase, could represent the evolutionarily conserved ortholog of the P69C subtilase from tomato, and the promoter activation was the clue for identifying the *csb3* mutant.

The *SBT3.3* gene encodes a 777 amino acid preproenzyme (Fig. 1B and Supplemental Figure S3) containing a N-terminal 25 amino acid signal peptide followed by an 86-amino acid propolypeptide (aa 26 to 111), and a 666-amino acid mature polypeptide with a predicted molecular weight of 71237 Da. The mature polypeptide comprises eight potential asparagine-linked glycosylation sites (NXS/T). On the basis of sequence similarities with other subtilases, including P69C [30], the amino acid residues Ser-555, Asp-145, and His-223 were identified as residues of the catalytic triad (Fig. 1B).

### Expression pattern of *SBT3.3* following pathogen inoculation

To mode of *SBT3.3* gene regulation in plant immunity was assessed by inoculating Col-0 leaves with the bacterial pathogen *Pseudomonas syringae* DC3000 (*Ps*DC3000), carrying or not the avirulence gene *AvrRpm1*, and temporal gene expression patterns were determined by quantitative RT-PCR (RT-qPCR). *SBT3.3* was barely detectable in mock-inoculated plants, but strongly induced during the *Ps*DC3000 immune response (Fig. 1C). However, inconsistent with observations for SA-regulated marker genes (*i.e. PR-1* and *PR-2*), *SBT3.3* induction was transient, peaking at 48 hpi (hours post inoculation) and abruptly decaying thereafter. The strongest induction was observed following inoculation with the avirulent strain *Ps*DC3000 (*AvrRpm1*) (Figure 1C); induction was again transient, peaking at 12 h.p.i, and decayed thereafter. *SBT3.3* expression preceded *PR-1* and *PR-2* gene induction, suggesting that the signals that set in motion transcriptional reprogramming of these two types of gene responses might differ. Expression of *PR-1* and *PR-2* genes in the distal non-inoculated leaves was also observed for *SBT3.3*, although distal expression was not as high as that attained in local leaves (Fig. 1C). High and rapid (within an hour) induced *SBT3.3* expression was also promoted by bacterial PAMP flg22 application to Col-0 plants (Supplemental Fig. S4), providing additional support for the association of *SBT3.3* expression with early innate immune response activation.

### SBT3.3 functions in disease resistance

**Figure 2:**
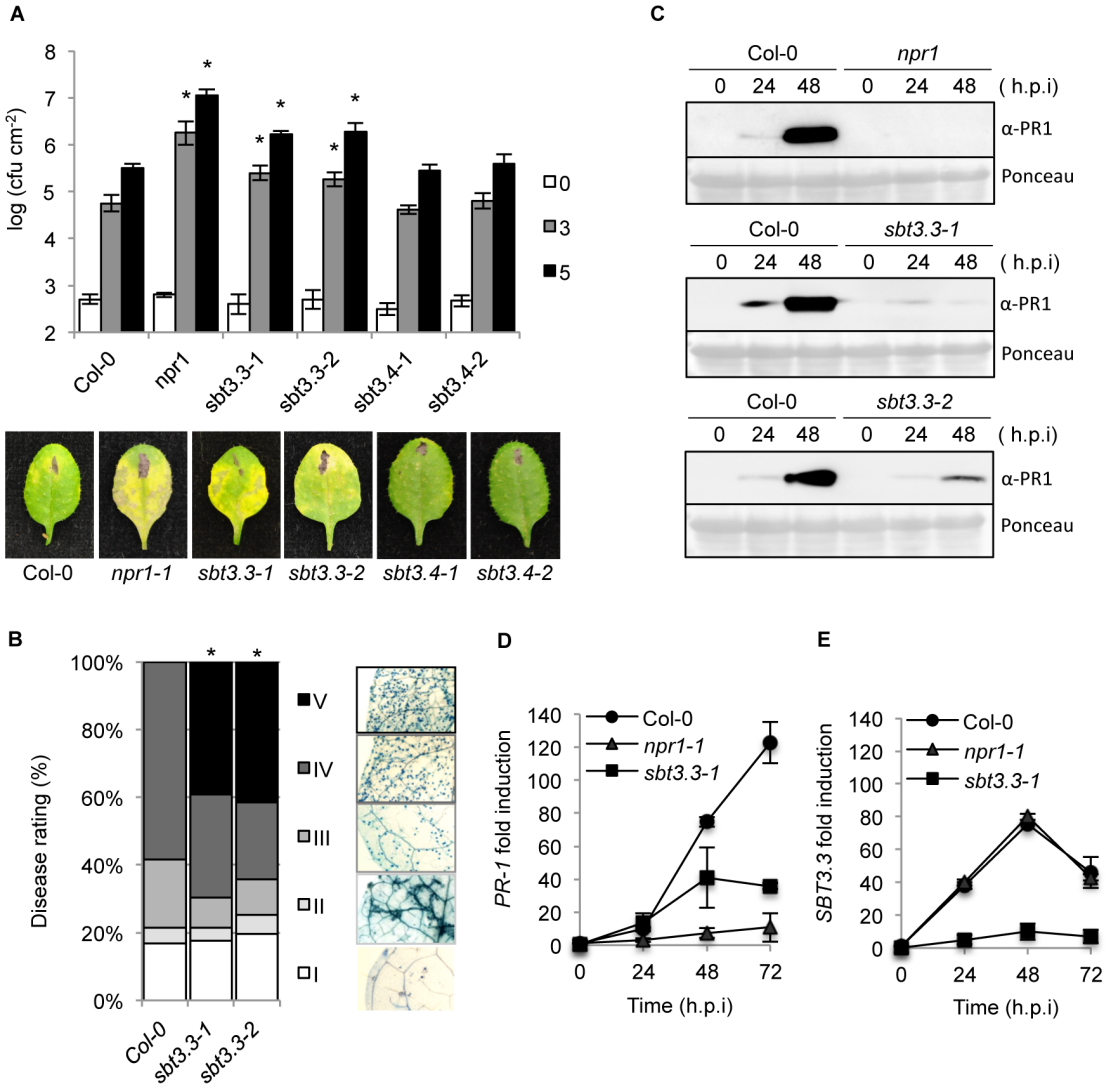
SBT3.3 loss of function increases disease susceptibility to *P.* *syringae* DC3000 and *H. arabidopsidis*. (**A**) Five-week-old plants were inoculated with *Ps*DC3000. Zero (white bars), three (grey bars) and five (black bars) days after inoculation, the bacterial growth was measured. Error bars represent standard deviation (n = 12). Asterisks indicate statistical differences to Col-0 (P<0.05) using Student's *t* test. Below are representatives of inoculated leaves of the indicated genotypes. (**B**) Quantification of *H. arabidopsidis* conidia development on Col-0, and *sbt3.3-1* and *sbt3.3-2* mutants. Asterisks indicate statistically different distributions of disease severity classes compared with Col-0 plants (χ^2^ test; α = 0.05). (**C**) Western blots with anti-PR1 antibodies reveals inhibition of PR1 induced accumulation in *nrp1*, *sbt3.3-1* and *sbt3.3-2* mutant plants, compared to Col-0, following inoculation with *Ps*DC3000. The experiments were repeated three times with similar results. (**D–E**) Time-course RT-qPCR analysis showing *PR-1* (D) and *SBT3.3* (E) gene expression in Col-0, *sbt3.3-1*, and *npr1-1* plants after infection with *Ps*DC3000. Data represent the mean ± SD; n = 3 replicates and gene expression given as in Fig. 1.

The importance of SBT3.3 in plant immunity was investigated by characterizing the response of two independent T-DNA insertion lines for *SBT3.3* (*sbt3.3-1* and *sbt3.3-2*; Fig. 1A) to *Ps*DC3000 infection (Fig. 2A). We also characterized the response of two independent T-DNA lines available for one of the linked subtilase genes (i.e. *SBT3.4*) within the same genomic cluster (*sbt3.4-1* and *sbt3.4-2*; Fig. 1A). The enhanced disease susceptibility mutant *npr1-1* was incorporated into the experiments as a control. Disease performance was assayed by measuring bacterial growth in the inoculated leaves (Fig. 2A). The two control mutant lines, *sbt3.4-1* and *sbt3.4-2* behaved as inoculated Col-0 plants, indicating that SBT3.4 is not essential to activate immune responses. However, *npr1* plants, and either one of the two *sbt3.3* mutants, supported significant increases in bacterial growth. The enhanced disease susceptibility was accompanied by development of disease symptoms in the form of visible chlorotic lesions on inoculated leaves (Fig. 2A). The results suggest that SBT3.3 positively regulates disease resistance to *Ps*DC3000.

Changes in the susceptibility of *sbt3.3* plants to biotrophic pathogens were further investigated by inoculating plants with a virulent strain of the obligate oomycete *Hyaloperonospora arabidopsidis* (isolate Noco) (Fig. 2B). Disease severity was assessed at 7 d.p.i in lactophenol trypan-blue-stained leaves. The leaves were classified into five categories (I to V) according to their degree of colonization by the oomycete (Fig. 2B). Both, *sbt3.3-1* and *sbt3.3-2* mutant plants exhibited a significantly higher degree of colonization by the oomycete than the control Col-0 plants (Fig. 2B), becoming heavily covered with sporangiophores, which elicited appearance of chlorosis and eventual leaf collapse (namely Class V). The observed enhanced disease susceptibility of *sbt3.3-1* and *sbt3.3-2* plants to *H. arabidopsidis* was corroborated by directly counting of spore production in inoculated plants (Supplemental Fig. S5). These results confirmed that loss of SBT3.3 function also enhanced plant susceptibility to *H. arabidopsisdis*, further substantiating its value in establishing an effective plant immune response.

### SBT3.3 is required for expression of SA-responsive genes

Compromised expression of SA-responsive genes is observed in mutants defective in resistance to biotrophic pathogens (i.e. *npr1*; [31]). Consequently, we considered the possibility that the increased susceptibility towards pathogens observed in SBT3.3 defective mutants might be similarly accompanied by a compromised expression of SA-responsive genes. Therefore, induction of PR-1 accumulation was examined by Western blot in *sbt3.3*, *npr1*, and Col-0 plants following inoculation with *Ps*DC3000. The PR-1 protein, as expected, was nearly absent in *npr1* plants, even at 48 hpi with *Ps*DC3000 (Fig. 2C), while PR-1 accumulation was notable in Col-0 following inoculation. Interestingly, *sbt3.3-1* and *sbt3.3-2* plants exhibited results similar to the *npr1* mutant, showing a notable impediment to induced PR-1 protein accumulation post pathogen inoculation (Fig. 2C). These results were confirmed at the transcriptional level by measuring *PR-1* transcript level by RT-qPCR (Fig. 2D). As for NPR1 being required for full immunity, our results suggest that SBT3.3 is required for full expression of downstream SA-responsive genes. This helps explaining why mutants defective in SBT3.3 are compromised in disease resistance (Figure 2A and B).

### *SBT3.3* expression is SA-independent and responds to H_2_O_2_

**Figure 3:**
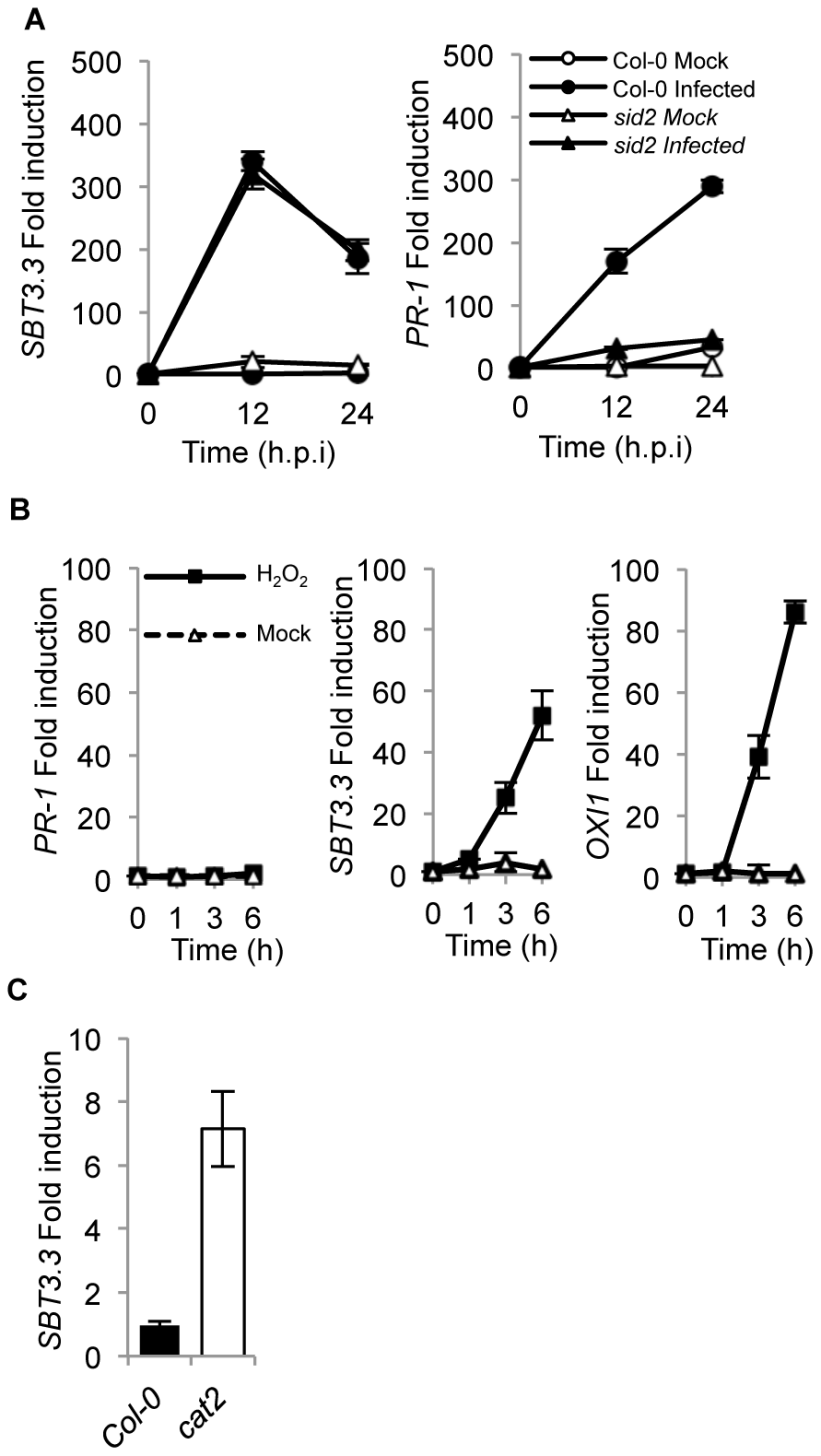
*SBT3.3* gene expression is SA-independent and rapidly induced by H_2_O_2_. (**A**) RT-qPCR analysis showing *SBT3.*3 gene expression in mock- (white symbols) and *Ps*DC3000 (*AvrRpm1*)-inoculated (black symbols) leaves in Col-0 (circles) and *sid2-1* (triangles). (**B**) RT-qPCR analysis showing *PR-*1, *SBT3.3* and *OXI1* gene expression in mock- (white triangles), and H_2_O_2_-treated (black squares) Col-0 seedlings. (**C**) *SBT3*.3 expression level in a *cat2* mutant. Data represent the mean ± SD; n = 3 replicates and gene expression given as in Fig. 1.

The same mRNA preparations shown in Figure 2D were used to quantify *SBT3.3* transcript accumulation following inoculation with *Ps*DC3000 in Col-0 and *npr1* plants (Fig. 2E). RNA preparations from *sbt3.3* plants (here used as a control) served to demonstrate that in the mutant induced *SBT3.3* expression was drastically down-regulated due to the T-DNA insertion. In marked contrast with the substantial reduction observed for *PR-1* activation (Figure 2D), *SBT3.3* expression in *npr1* plants was identical to that attained in Col-0 plants. Furthermore, in *sid2-1* mutant plants (defective in SA synthesis) induction of *SBT3.3* expression upon inoculation with *Ps*DC3000(*AvrRpm1*), remained unchanged with respect to Col-0 plants (Fig. 3A). This differs with the compromised expression of *PR-1* occurring in *sid2* plants (Fig. 3A). These contrasting differences indicated that for pathogen-induced *SBT3.3* expression, SA synthesis and its perception through NPR1 are dispensable.

Since oxidative burst and concurrent H_2_O_2_ accumulation preceded SA build-up during basal immunity activation [32] and *SBT3.3* induction appeared as an early event preceding *PR* gene induction by SA (Fig. 1C–D), we hypothesized that H_2_O_2_ could mediate *SBT3.3* induction. In fact, spraying Arabidopsis leaves with a 1 mM solution of H_2_O_2_ elicited a rapid *SBT3.3* induction which was notable at 1 to 3 hours after treatment (Fig. 3B). Similarly, expression of the *OXI1* gene, which encodes a kinase highly induced under oxidative stress conditions [33] was triggered by H_2_O_2_ (Fig. 3B). However, under similar inductive conditions expression of the SA-regulated gene *PR-1* remained unchanged (Fig. 3B). Moreover, the *cat2* mutant defective in the dismutation of H_2_O_2_ and exhibiting enhanced H_2_O_2_ accumulation, revealed increased *SBT3.3* expression compared to Col-0 (Fig. 3C). These observations indicated that *SBT3.3* activation might result from early H_2_O_2_ production during the immune response.

### SBT3.3 is secreted and accumulates extracellularly

**Figure 4:**
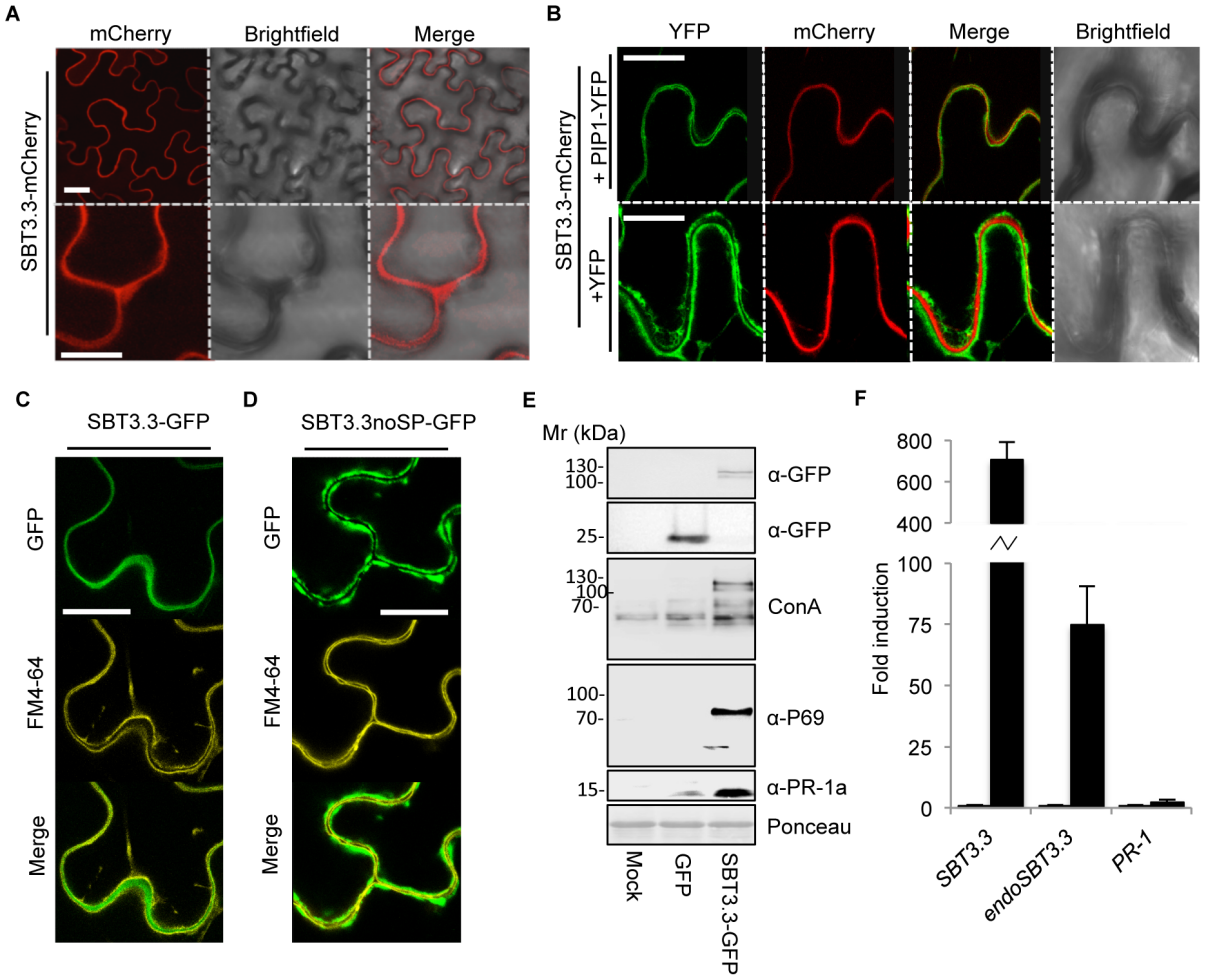
Extracellular localization of SBT3.3-mCherry in *N.* *benthamiana* leaves by confocal microscopy. (**A**) Expression of SBT3.3-mCherry (50 hpi) results in a uniform extracellular fluorescence. (**B**) Co-expression of SBT3.3-mCherry with the plasma membrane marker PIP-YFP (upper panel) and with free cytosolic YFP (lower panel). (**C**) Co-localization of SBT3.3-GFP with the plasma membrane fluorescent marker FM4-64. (**D**) Co-localization of SBT3.3noSP-GFP with the plasma membrane fluorescent marker FM4-64. Scale bars are 10 µm (**A**-upper panel), 40 µn (**A**-lower panel, B, C and D panels). (**E**) Western blots of total protein extracts from *N. benthamiana* leaves transitorily overexpressing GFP alone or a SBT3.3-GFP fusion proteins revealed with either concanavalin A, or with anti-GFP, anti-P69 and anti-PR-1a antibodies. Total protein extracts from empty *A. tumefaciens* agroinfiltrated *N. benthamiana* leaves (mock) were used as controls. (**F**) Overexpression of *SBT3.3* under the control of a *35S* promoter in stable transgenic Arabidopsis plants triggers activation of the endogenous *SBT3.3* gene. Accumulation levels of the 35S driven *SBT3.3* transcripts (*SBT3.3*), the endogenous *SBT3.3* transcripts (endo*SBT3.3*) and *PR-1* transcripts were measured comparatively in healthy Col-0 plants (left bars) and in a transgenic *35S::SBT3.3OEX* line (right black bars). Data represent the mean ± SD; n = 3 biological replicates and gene expression given as in Fig. 1.

Plant subtilases are synthesized in the form of preproprotein precursors, translocated via a signal peptide into the endomembrane system, and activated through further cleavage of the propeptide [34]. Most plant subtilases are considered glycoproteins that predominantly accumulate extracellularly [30], [34], [35]. SBT3.3 subcellular localization was determined by fusing monomeric cherry fluorescent protein (mCherry) to the C-terminus of the full length SBT3.3, and the construct, driven by 35S promoter, expressed in *Nicotiana benthaminana* leaves using agro-infiltration. Localization of the fusion protein was confirmed by confocal microscopy. Results showed that SBT3.3-mCherry fluorescence was uniformly distributed in the pericellular apoplastic space (Fig. 4A). Similar pericellular localization was observed in transgenic Arabidopsis plants expressing a *35S::SBT3.3-GFP* gene construct (Supplemental Fig. S6). The mCherry-tagged subtilase was co-expressed with either a construct bearing the plasma membrane integral protein PIP1 fused to YFP, or alternatively with a free cytosolic YFP protein to more precisely define its localization. SBT3.3-mCherry was localized externally to the cytoplasm, as revealed when co-expressed with a free cytosolic YFP (Fig. 4B). Furthermore, SBT3.3-mCherry was found to be sandwiched between the PIP1-YFP-tagged plasma membrane marker of adjacent cells (Fig. 4B) and thus unambiguously localized to the extracellular matrix. SBT3.3 extracellular localization was also confirmed upon expression of a SBT3.3-GFP protein fusion in the presence of FM4-64, a plasma membrane specific fluorescent dye (Fig. 4C). Furthermore, expression of an *SBT3.3noSP-GFP* gene construct in which the N-terminal 25 amino acid signal peptide of SBT3.3 has been deleted, revealed that the SBT3.3noSP-GFP protein fusion was not secreted to the extracellular matrix and was retained in the cytoplasm, as delineated by the co-localization with FM4-64 (Fig. 4D). These results indicate that SBT3.3 is secreted and accumulated in the plant extracellular matrix, and secretion depends on the presence of its signal peptide.

### SBT3.3 expression in *N. benthamiana* activates expression of an endogenous P69 subtilase

Chichkova *et al.* [36] demonstrated that following agro-infiltration with a GFP-tagged phytaspase, an Arabidopsis cell death-associated subtilase of similar size to SBT3.3; two proteins of ∼110 and ∼120 kD corresponding to the mature and the pro-protein phytaspases, respectively, accumulated in crude extracts [34]. Following expression of SBT3.3-GFP, two similar protein bands of ∼110 and ∼120 kD were detected in Western blots using an anti-GFP antibody (upper panel; Fig. 4E). The difference between the theoretical mature 98,2 kD SBT3.3-GFP fusion and the observed mature 110 kD proteins must be due to posttranslational modifications. In fact, glycosylation was early proposed to regulate activity of plant subtilases [37], [38]. We performed Western blots of the same leaf extracts and developed the nitrocellulose filters with Concanavalin A (Con A) coupled to horseradish peroxidase to identify if SBT3.3 is glycosylated. These analyses revealed that Con A recognized the 120/110 kD doublet (Fig. 4E) in extracts expressing the *SBT3.3-GFP* construct, thus confirming that SBT3.3 is glycosylated. Interestingly, Con A also recognized a band of ∼70 kD that only accumulated following SBT3.3-GFP expression (Figure 4E). This ∼70 kD band was reminiscent of a glycosylated 69 kD P69 subtilase conserved in the Solanaceous species [27], [30]. To verify this possibility, Western blots were developed with anti-P69C antibodies [30]. Results revealed that the ∼70 kD Con-A reacting band was recognized by anti-P69 antibodies (Fig. 4E) indicating that SBT3.3-GFP signaled tobacco cells to activate expression of an endogenous P69 subtilase homologue. Interestingly, when we extended this analysis to other defense-related proteins, i.e. by using antibodies against the PR-1a isoform from tobacco, we observed that overexpression of SBT3.3-GFP similarly triggered SA-responsive PR-1a protein accumulation (lower panel; Fig. 4E). We subsequently created a missense mutant in the SBT3.3-GFP “catalytic triad” (S555A; SBT3.3m-GFP), to ascertain if the observed signaling required integrity of the subtilase proteolytic activity. Expression of the missense mutant in *N. benthamiana* no longer promoted local accumulation of the corresponding endogenous P69 subtilase, or the PR-1a protein (Supplemental Figure S7). All of these observations are consistent with a model in which Arabidopsis SBT3.3 subtilase local expression autonomously triggers immune-like responses in a heterologous system, and the serine proteolytic activity of the subtilase is necessary for this effect. As occurs in *SBT3.3OEX1* plants (see below), expression of the *SBT3.3-GFP* gene construct in transgenic Arabidopsis plants conferred enhanced disease resistance to *Ps*DC3000 and is in contrast with the lack of effect observed for the *SBT3.3m-GFP* construct (Supplemental Figure S8). Moreover, the same *SBT3.3-GFP* gene construct is able to abrogate the characteristic enhanced disease susceptibility phenotype of *sbt3.3* plants, conferring enhanced disease resistance to *Ps*DC3000 to the stably transformed *sbt3.3* mutant plants (Supplemental Figure S9). This further indicates functionality of SBT3.3-GFP fusion protein in promoting immune responses in Arabidopsis.

### *SBT3.3* artificial expression in transgenic Arabidopsis triggers expression of the endogenous gene

The above observations indicated that SBT3.3 promotes the expression and accumulation of a homologous subtilase (i.e. P69) in N. benthamiana. We studied a stable transgenic Arabidopsis line constitutively expressing SBT3.3 under the control of the 35S promoter (SBT3.3OEX) by measuring activation on the corresponding endogenous SBT3.3 gene to test if the same phenomenon could be reproduced in Arabidopsis. Antibodies against SBT3.3 were not available; therefore we instead performed RT-qPCR measurements using 2 different pairs of oligonucleotides. One of those pairs discriminates between the endogenous *SBT3.3* mRNAs (*endoSBT3.3*), transcribed from its own gene, and the other pair was designed to measure the whole amount of *SBT3.3* mRNAs (*SBT3.3*). In Col-0 plants, as expected, both *SBT3.3* and *endoSBT3.3* transcript expression was very low (Fig. 4F). In contrast, in the transgenic *SBT3.3OEX* line, *SBT3.3* transcript accumulation was prominent (Fig. 4F), and importantly, this was also followed by a high *endoSBT3.3* transcript accumulation. This effect gives support to the hypothesis that *SBT3.3* expression is able *per se* to signal its own gene activation. This induction appears “self” controlled, since endogenous *PR-1* transcript levels in transgenic plants do not exhibit significant variation with respect to Col-0 plants (Fig. 4F). However, in agro-infiltrated tobacco leaves we observed that *SBT3.3-GFP* expression promotes accumulation of both the endogenous P69 subtilase homolog, and the SA-dependent PR-1a protein. This difference can only be explained by noting that in the experiments with *N. benthamiana*, *A. tumefaciens* is inevitably present, which in turn may supply PAMPs in collaboration with SBT3.3, triggering a downstream SA signaling pathway, and in turn SA-defense related gene activation. If this mechanism operated effectively, then SBT3.3 would be required to facilitate early immune signaling preceding defense response activation.

### *SBT3.3* overexpression confers enhanced disease resistance to *Ps*DC3000 and *H. arabidopsidis*

**Figure 5:**
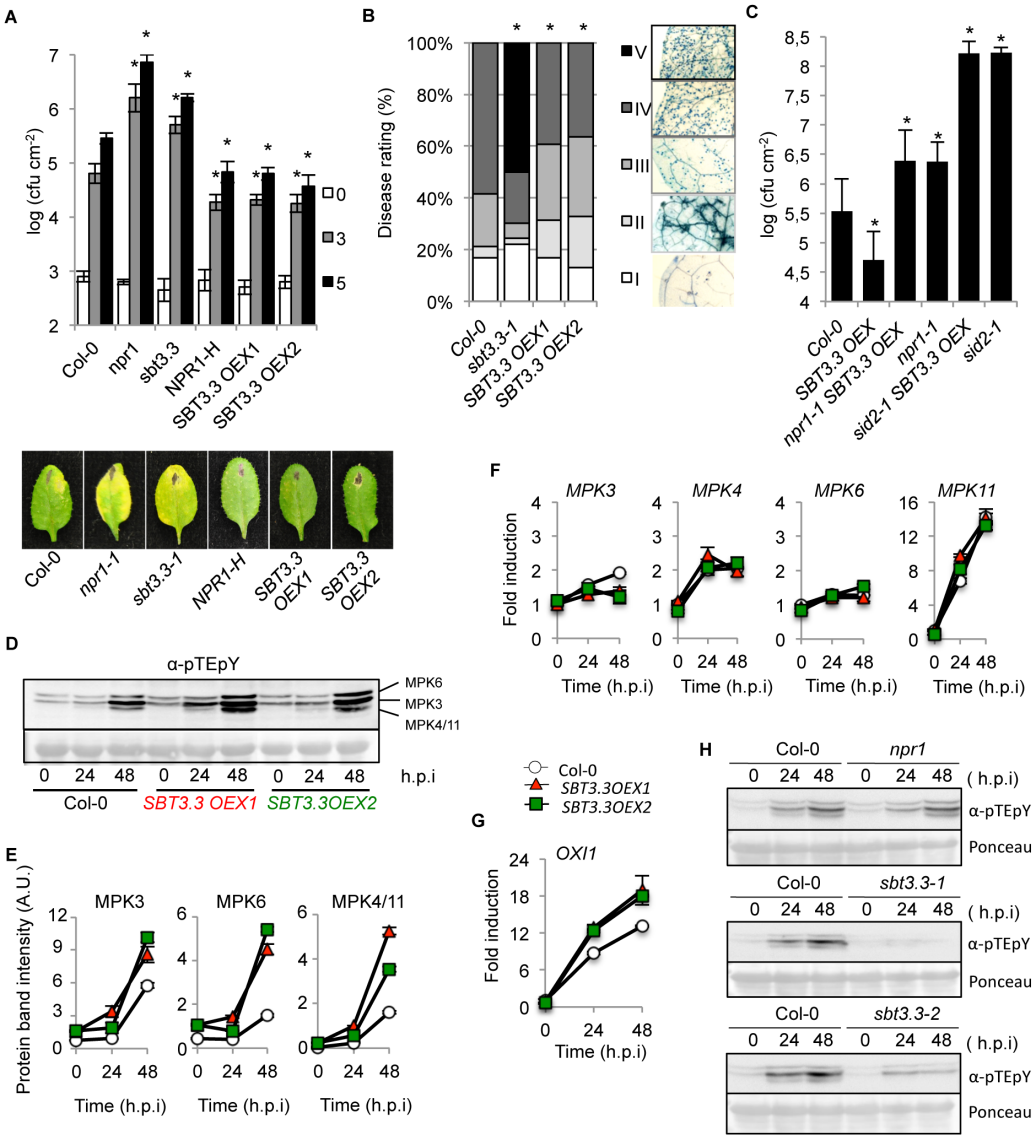
SBT3.3 overexpression confers enhanced disease resistance and enhanced mitogen-activated kinase activation. (**A**) Plants of the indicated genotype were inoculated with *Ps*DC3000. Zero (white bars), three (grey bars) and five (black bars) days after inoculation, the bacterial growth was measured. Error bars represent standard deviation (n = 12). Asterisks indicate statistical differences to Col-0 (P<0.05) analysed with a Student's *t*-test. Below are shown representative pictures of leaves of the inoculated plants genotypes. (**B**) Quantified *H. arabidopsidis* conidia development on leaves of the indicated genotypes. Asterisks indicate statistically different distributions of disease severity classes compared with Col-0 plants (χ^2^ test; α = 0.05). (**C**) Disease resistance phenotype of homozygous double *npr1 SBT3.3OEX1* and *sid2 SBT3.3OEX1* mutant plants against *Ps*DC3000 was compared to Col-0 and to their respective parental lines. Experiments were performed as described in Figure 2A. (**D**) Western blot with anti-pTEpY antibodies of crude protein extracts derived from Col-0, *SBT3.3OEX1* and *SBT3.3OEX2* plants at 0, 24, and 48 h.p.i with *Ps*DC3000. Equal protein loading was check by Ponceau staining of the nitrocellulose filter. MAP6, MPK3 and MPK4/11 migrating bands are indicated on the right. The experiments were repeated three times with similar results. (**E**) Densitometric scan quantification of protein bands corresponding to MPK3, MPK6 and MPK4/11 bands as shown in (D) following inoculation of Col-0 (white), SBT3.3OEX1 (red) and SBT3.3OEX2 (green) plants with *Ps*DC3000. Data represent the mean ± SD; n = 3 replicates. (**F–G**) Time-course RT-qPCR analysis showing *MPK3*, *MPK4*, *MPK6*, *MPK11* (**F**) and *OXI1* (**G**) gene expression in the indicated genotypes following inoculation with *Ps*DC3000. Data represent the mean ± SD; n = 3 replicates. Expression was normalized to the constitutive *ACT2* gene expression as in Fig. 1. (**H**). Western blot with anti-pTEpY antibodies of crude protein extracts derived from Col-0, *npr1, sbt3.3-1 and sbt3.3-2* plants at 0, 24, and 48 h.p.i with *Ps*DC3000. Equal protein loading was check by Ponceau staining of the nitrocellulose filter. The experiments were repeated three times with similar results.

Two independent Arabidopsis transgenic lines that overexpress *SBT3.3* were inoculated with *Ps*DC3000 and disease response recorded to further assess the role of SBT3.3 subtilase in plant immunity (*i.e. SBT3.3OEX1* and *SBT3.3OEX2*). The enhanced disease resistance line overexpressing the NPR1 regulator (*NPR1-H*, [39]), one of the SBT3.3 defective mutants (*i.e. sbt3.3-1*), and Col-0 were included in this experiment for comparison. Figure 5A shows that the two *SBT3.3OEX* lines exhibited significant enhanced disease resistance responses to *Ps*DC3000 compared to Col-0. This enhanced resistance was of a magnitude similar to that attained by *NPR1-H* plants. In contrast, the *sbt3.3-1* mutant reproduced the expected increased disease susceptibility previously shown in Figure 2.

The SBT3.3 overexpressing lines exhibited an enhanced disease resistance phenotype when exposed to *H. arabidopsidis* (Fig. 5B). The two lines overexpressing SBT3.3 exhibited a significantly lower colonization of the oomycete than the control Col-0 plants or the highly susceptible *sbt3.3-1* mutant (Fig. 5B). The observed enhanced disease resistance of *sbt3.3-1* to *H. arabidopsidis* was corroborated by directly counting of spore production in inoculated plants (Supplemental Fig. S5). The observed heightened resistance against these two pathogens indicated that SBT3.3 functions as a positive plant immunity regulator. Furthermore, when the *SBT3.3* overexpression phenotype was assayed in an *nrp1* or *sid2* mutant background, the enhanced disease resistance to *Ps*DC3000 was abrogated (Fig. 5C). These results indicate that SBT3.3, as a positive plant immunity regulator, operates upstream of the SA pathway.

### *SBT3.3* expression confers enhanced activation of MPK kinases

Elevated mitogen-activated protein kinases (MAPKs) activation is genuinely linked to IR development [12], and in general to innate immune responses [40], [41], [42]. Therefore, our next objective was to demonstrate if the enhanced resistance phenotype mediated by the sole SBT3.3 subtilase expression could elicit elevated MPKs activation. We subsequently employed an antibody recognizing the phosphorylated residues within the MAPK activation loop (the so called *p*TE*p*Y motif, where *p* denotes the phosphorylated residue). Western blot analysis of protein extracts derived from healthy Col-0 plants or from two *SBT3.3OEX* lines revealed positive immunoreactive signals in two polypeptides corresponding to MPK6 and MPK3 [42]. Following densitometric scanning of Western blots, the two immunoreactive bands appeared moderately more intense in the overexpression lines relative to Col-0 control lines (Fig. 5D–E). Inoculation with *Ps*DC3000 promoted a further activation-associated dual TEY phosphorylation of MPK3 and MPK6, which was higher in the two *SBT3.3OEX* lines than in Col-0 plants (Fig. 5D–E). In addition, MPK4/MPK11, which migrated as a single band on SDS-PAGE [42], became activated only following bacterial inoculation, and activation was again more intense in the two *SBT3.3OEX* lines. Therefore, dual phosphorylation of the TEY amino acid motif within the MPK activation loop, which is required for kinase activity appeared increased in plants expressing SBT3.3. However, despite these differences at the protein level, no significant differences were detected with respect to transcript accumulation induction for these MPKs between Col-0 and *SBT3.3OEX* lines (Fig. 5F).

OXI1 is a serine/threonine kinase of the AGC protein kinase family required for oxidative burst-mediated signaling in Arabidopsis; its expression was consistent with that of *SBT3.3*, and was induced by H_2_O_2_ ([33]; and Fig. 3B). OXI1 was required for MPK3 and MPK6 activation and for basal resistance to *H. arabidopsidis* [33]. In view of these observations, we hypothesized that the imposed *SBT3.3* expression might sensitize cells to bring earlier or higher *OXI1* expression levels following pathogen infection, and in turn provide an explanation for the higher activation observed in MPKs. We measured *OXI1* comparative transcript level between Col-0 and two *SBT3.3OEX* lines following *Ps*DC3000 infection by RT-qPCR. Results indicated the two *SBT3.3OEX* lines expressed *OXI1* to higher levels than Col-0 (Fig. 5G). This offers a viable explanation as to why MPKs exhibited increased activation in *SBT3.3OEX* plants following pathogen attack, even in the absence of differential gene expression relative to Col-0. Moreover, in *sbt3.3* mutant lines MPKs activation following inoculation with *Ps*DC3000 was drastically reduced in comparison to Col-0 (Figure 5H). This observation reinforces the consideration that SBT3.3 appears to function as a positive regulator of the pathway leading to activation of MAP kinases. Interestingly, MPKs activation was not altered in the enhanced disease susceptibility *nrp1* mutant (Figure 5H) and neither was the expression of the *SBT3.3* gene altered in this same mutant (Figure 3F). This served as a control towards the specific requirement of SBT3.3 for MAKs activation and suggest that this specific signal module operates upstream of the NPR1 regulator.

### SA-mediated defense genes are poised for enhanced activation in plants overexpressing *SBT3.3*

**Figure 6:**
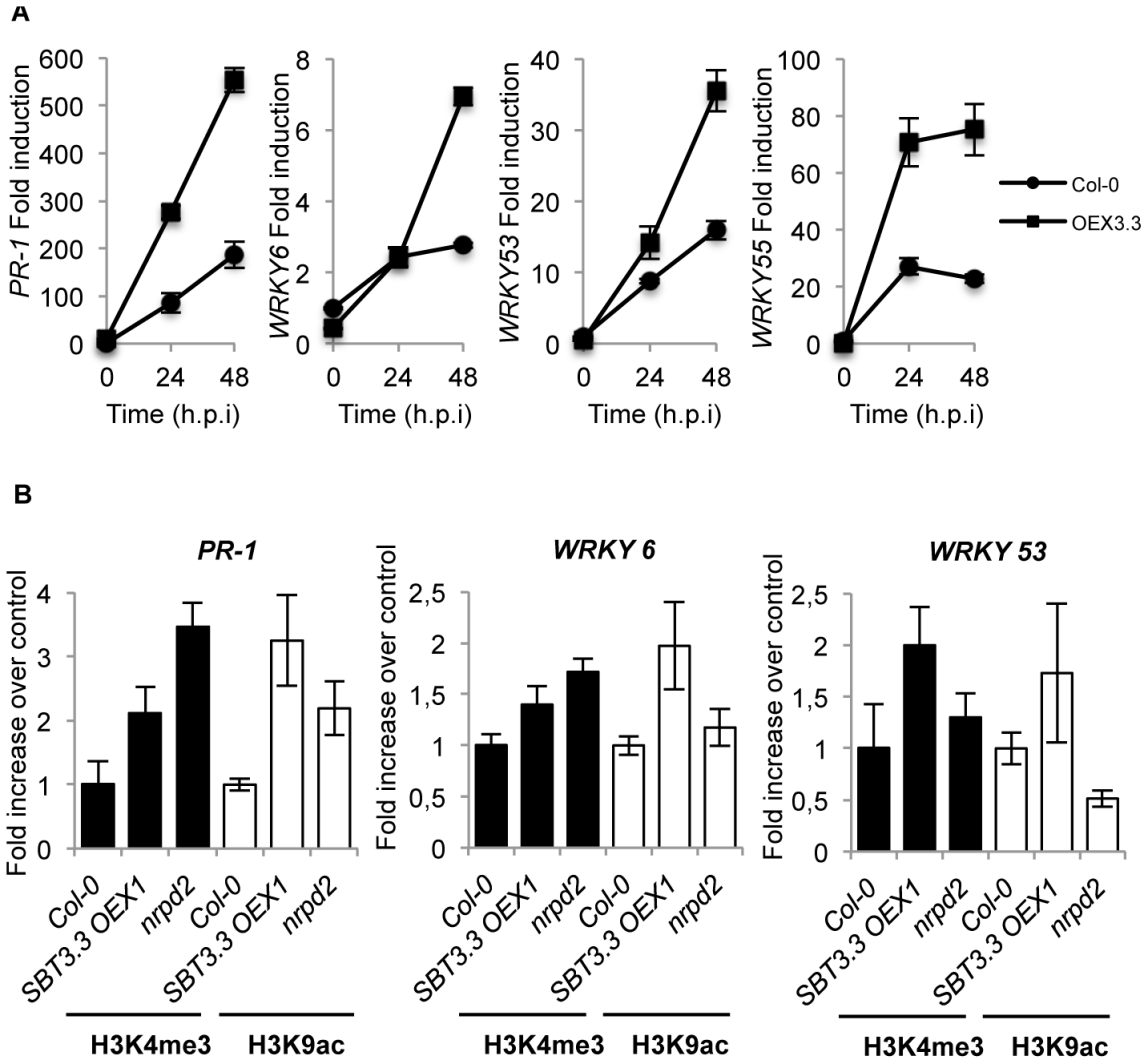
SBT3.3 expression poises SA-mediated defense genes for enhanced activation. (**A**) RT-qPCR of *PR-1, WRKY6, WRKY53 and WRKY55* transcript levels following inoculation with *Ps*DC3000 in Col-0 and *SBT3.3OEX1* plants. Data represent the mean ± SD; n = 3 replicates. (**B**) Comparative level of histone H3 Lys4 trimethylation (H3K4me3) and histone H3 K9 acetylation (H3K9ac) on the *PR-1*, *WRKY6* and *WRKY53* gene promoters as present in leaf samples from Col-0, *SBT3.3OEX1* and *nrpd2* plants. Data are standardized for Col-0 histone modification levels. Data represent the mean ± SD; n = 3 biological replicates. Expression was normalized to the expression of the constitutive ACT2 gene.

The above results prompted us to search if SA-dependent genes were poised for increase activation following *SBT3.3* expression. Therefore, we inoculated Col-0 plants, and one *SBT3.3OEX* line with *Ps*DC3000, and compared *PR-1* expression patterns. Interestingly, after pathogen inoculation induction of *PR-1* gene expression showed a notorious enhancement in *SBT3.3OEX* plants when compared to Col-0 (Fig. 6A). Moreover, the genes encoding the transcription factors WRKY6, WRKY53, and WRKY35, mediating transcriptional regulation of SA-related genes, including *PR-1* [43], themselves induced by pathogen infection [44], showed similar enhanced induced expression in *SBT3.3OEX* plants (Fig. 6A). Thus, SBT3.3 mediated poising of defense genes for enhanced activation following perception of a pathogenic cue, invoking a role for SBT3.3 in priming immune responses.

Poising SA–related genes and primed immunity concur in plants defective in the RdDM pathway, such as mutants affected in different subunits of the RNA Pol V (*i.e. nrpd2*) [23], and also following pharmacological treatment with the priming agent BTH [20]. In both cases, chromatin histone activation marks appeared enriched in SA-related gene promoters. Consequently, we hypothesized that following expression of *SBT3.3* SA-related defense genes could be poised for enhanced activation by differential histone modification. By using chromatin immunoprecipitation (ChIP), we analyzed H3 Lys4 trimethylation (H3K4me3) and H3 Lys9 acetylation (H3K9ac) on the *PR-1*, *WRKY6* and *WRKY53* gene promoter region in both Col-0 plants and *SBT3.3OEX* plants. The enhanced disease resistant mutant *nrpd2*, defective in RNA PolV activity and compromised in the RdDM pathway [23], was included as a control. On the *PR-1* promoter, H3K4me3 and H3K9ac activation marks increased more than two and three-fold, respectively in *SBT3.3* overexpressing plants when compared to Col-0 (Fig. 6B). The two activation marks were similarly increased in *nrpd2* plants, with only some differences in intensity (Fig. 6B). As for *PR-1*, the histone marks also showed increases in the *WRKY6* and *WRKY53* promoters in *SBT3.3* overexpressing plants, and to a lesser extent in *nrpd2* plants, relative to Col-0 plants (Fig. 6B). Therefore, chromatin marks normally associated with active genes abound in the promoter regions of SA-related genes in SBT3.3 overexpressing plants, although gene activation does not occur in these plants. The marks appear to serve as an on-switch for priming, and helps explain why the same genes show enhanced induction in *SBT3.3OEX* plants upon pathogenic attack (Fig. 6A).

### SBT3.3 expression increases H3K4me3 activation marks in its own promoter

**Figure 7:**
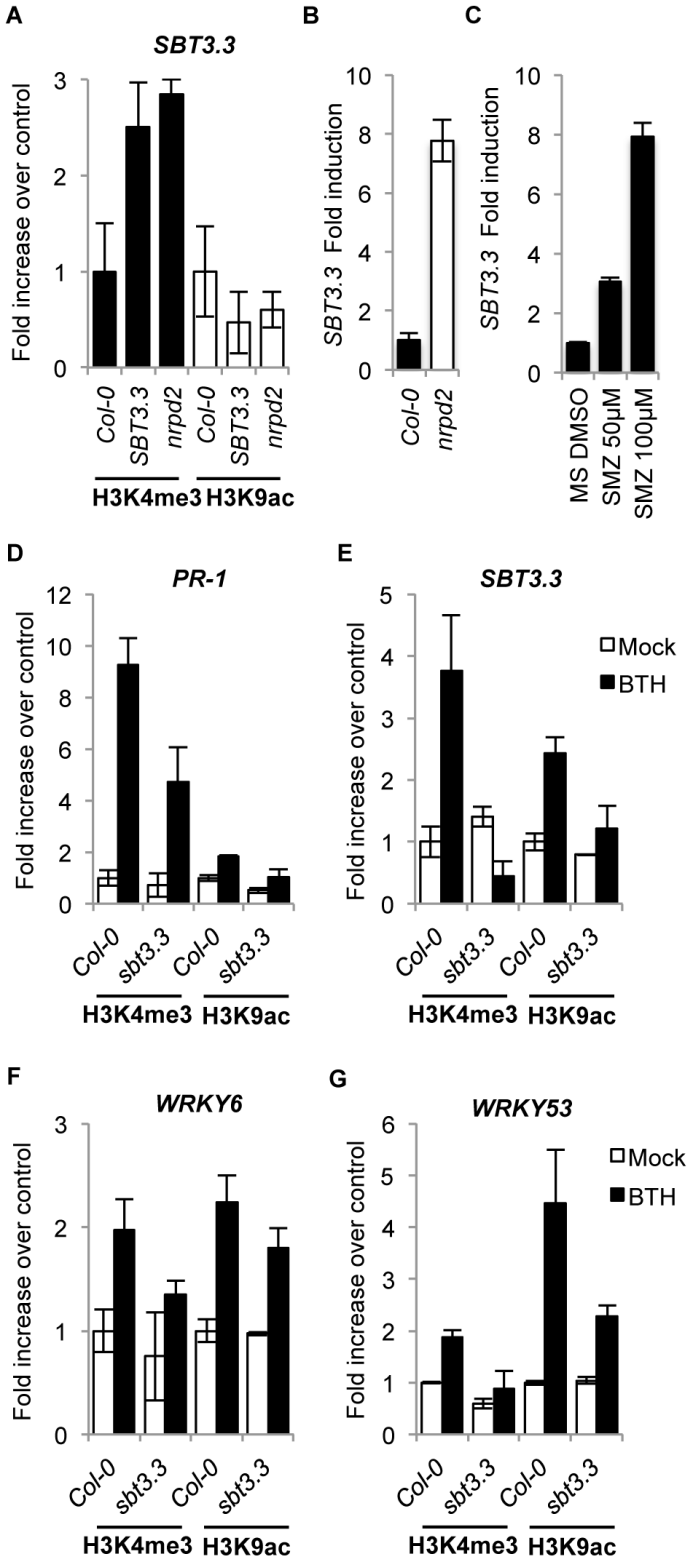
SBT3.3 expression promotes chromatin remodeling and is under epigenetic control. (**A**) Comparative level of H3K4me3 and H3K9ac mark setting on the *SBT3.3* gene promoter as present in leaf samples from Col-0, *SBT3.3OEX1* and *nrpd2* plants. Data are standardized for Col-0 histone modification levels. Data represent the mean ± SD; n = 3 biological replicates. (**B**) RT-qPCR of *SBT3.3* transcript levels in Col-0 and *nrpd2* plants. Data represent the mean ± SD; n = 3 biological replicates. Expression was normalized to the expression of the constitutive *ACT2* gene and then to the expression in Col-0 plants. (**C**) RT-qPCR of *SBT3.3* transcript levels in Col-0 seedlings upon treatment with 50 µM and 100 µM of sulfamethazine (SMZ) compared to mock (DMSO). Data represent the mean ± SD; n = 3 biological replicates. Expression was normalized to the expression of the constitutive *ACT2* gene, then to mocked Col-0 plant expression. (**D–G**) Comparative level of H3K4me3 and H3K9ac mark setting on *PR-1* (**D**), *SBT3.3* (**E**), *WRKY6* (**F**) and *WRKY53* (**G**) gene promoters in Col0 and *sbt3.3* plants following treatment with 100 µM BTH compared to buffer alone (mock). Data represent the mean ± SD; n = 3 biological replicates.

Results showed the sole expression of *SBT3.3* in transgenic Arabidopsis plants was able to promote activation of the endogenous gene (Figure 4F). Therefore, SBT3.3 *per se* might be signaling chromatin remodeling of its own promoter as it does for the *PR-1* gene promoter (Fig. 6B). ChIP for H3K4me3 and H3K9ac marks at the *SBT3.3* promoter region in *SBT3.3* overexpressing plants revealed that the H3K4me3 mark was notably increased compared to Col-0 plants, and moreover, the enhancement in H3K4me3 marks was mirrored in *nrpd2* plants (Fig. 7A). However, H3K9ac marks in the *SBT3.3* gene promoter did not increase in the *SBT3.3* overexpressing plants and the *nrpd2* mutant when compared to Col-0 (Fig. 7A). These results contrasted with the common increase of both activation marks in the *PR-1* gene (Fig. 6B), and suggested the existence of specific histone codes regulating gene expression. Alternatively, because the increase in H3K4me3 activation marks observed in the *SBT3.3* gene promoter between *nrpd2* and *SBT3.3OEX* plants were matched, we reasoned that *nrpd2* plants might also carry constitutive *SBT3.3* gene expression. *SBT3.3* transcript level determination by RT-qPCR in Col-0 and *nrpd2* plants showed the mutant constitutively expressing *SBT3.3* (Fig. 7B). These results suggest that *SBT3.3* expression is under negative epigenetic control, and expression is relieved following inhibition of RdDM. In fact, treatment of Col-0 seedlings with sulfamethazine (SMZ), a chemical suppressor of epigenetic gene silencing (i.e. RdDM) that derepress silenced genes [45], relieved *SBT3.3* and promoted transcript accumulation (Fig. 7C), to levels similar to those attained in *nrpd2* plants. These observations therefore support the contention of an epigenetic control towards *SBT3.3*, and indicate that SBT3.3 acts as a positive regulator of a priming phenomenon for more efficient deployment of immune responses. In addition, a low concentration (100 µM) pharmacological treatment with of the priming agent BTH administered to Col-0 plants promoted the enhanced deposition of H3K4me3 and H3K9ac marks in the *PR-1* gene promoter, and to a minor extent also in *WRKY6* and *WRKY53* gene promoters (Fig. 7D), as was observed in previous studies [20], [23]. Similarly, BTH also induced H3K4me3 activation marks, and to a less extent also of H3K9ac marks, in the *SBT3.3* gene promoter in Col-0 plants. Thus, as a priming agent, BTH not only induced chromatin remodeling of SA-related genes similar to the RdDM-defective and enhanced resistance mutant *nrpd2*; it also mimicked *SBT3.3* chromatin remodeling triggered by the SBT3.3 itself.

The importance of SBT3.3 in mediating activation of chromatin remodeling during priming induction was further evaluated in SBT3.3 defective plants. Figure 7D, F and G show that BTH-mediated increases of H3K4me3 and H3K9ac activation marks in the *PR-1, WRKY6* and *WRKY53* gene promoters was partially impeded in *sbt3.3* plant when compared to Col-0 plants. For the *SBT3.3* gene promoter (Fig. 7E), the reduction in histone activation marks following BTH treatment of *sbt3.3* plants was most dramatic. This severe reduction was most notorious in the case of H3K4me3 activation marks for which a full inhibition was observed in BTH-treated *sbt3.3* plants when compared to BTH-treated Col-0 plants (Figure 7D). All the above observations therefore imply that the extracellular SBT3.3 subtilase is an integral component mediating establishment of primed immunity, and moreover, it appears to be targeted for negative epigenetic control of this same mechanism.

## Discussion

Priming, an evolutionarily conserved phenomenon where cells respond to much lower levels of a pathogenic stimulus in a more rapid and robust manner, is an important component of the various forms of IR described in mammals [8]–[10], plants [6], [18] and invertebrates [11]. Despite the importance of priming, the signal component(s) mediating this sensitized state remain elusive. The sensitized state is in part explained by presumably dormant or silent component characters, which accumulate during priming, and are required only after pathogenic challenge [18]. In this respect, pre-stress deposition of two MPK family members of signaling enzymes, MPK3 and MPK6, has been described to play an important role for priming in Arabidopsis [12]. However, it remains undetermined whether or not activation of additional factors operating upstream of the two MPKs is required to establish priming.

Reverse genetic analysis was applied to identify the Arabidospsis *SBT3.3* gene, encoding a serine protease of the subtilisin clan, which is pivotal in control of a priming mechanism that leads to sensitization for activation of SA-dependent defense responses and IR. *SBT3.3* overexpression in transgenic Arabidopsis plants elicited enhanced disease resistance to pathogens. However, this enhanced resistance was not preceded by a high constitutive expression of SA-responsive genes as occurs in different disease resistant mutants, which in general carry associated dwarfism, such as in *cpr1*, *edr1* or *csb3* [26, and references therein] to mention a few. Instead, the SBT3.3-mediated resistance can be explained by accelerated and heightened activation of SA-responsive genes, only elicited by pathogen inoculation, therefore mimicking a wild type plant activated for priming. This phenotype is blocked in a *sid2* mutant background, which lacks SA, and is also blocked in the *npr1* mutant affected in signaling downstream of SA. SBT3.3 thus functions as a positive regulator of innate immunity operating upstream of the SA pathway. Consistent with the gain-of-function phenotype, *SBT3.3* suppression impairs induction of SA-responsive genes and causes enhanced susceptibility to infection by pathogens.

Interestingly, *SBT3.3* expression is rapidly demanded during activation of innate immunity preceding the activation of SA-responsive genes. However, in contrast to *PR* genes, *SBT3.3* expression does not require the SA pathway through the NPR1 regulator. Moreover, *SBT3.3* activation responds very rapidly to H_2_O_2_, a common ROS species generated very early during PAMP recognition by PRR leading to activation of innate immune responses. Consistent with other early induced and SA-independent genes [46], H_2_O_2_ might be the first signal for early transcriptional reprogramming of *SBT3.3*. Congruently, Daudi *et al.* [47] showed that knocking down the Arabidopsis cell wall peroxidases PRX33/PRX34, required for apoplastic H_2_O_2_ generation during innate immune responses, leads to changes in the cell wall proteome with depletion of various PAMP-elicited proteins, among which SBT3.3 was conspicuous [48]. Localized *SBT3.3* expression in the heterologous *N. benthamiana* system led to expression of the endogenous P69 homologue. Similarly, its stable expression in transgenic plants also led to activation of the endogenous *SBT3.3* gene by what appears to be a self-induction mechanism, which adds further novelty to the *SBT3.3* activation mode. We hypothesize that to a subsequent initial activation by a pathogen, the expressed SBT3.3 subtilase could initiate a signaling process, which would lead to its own expression, at least to a certain threshold level, as if forming a regulatory positive feedback loop circuit. Maintenance of this expression threshold level should be sufficient to keep cells in a sustained sensitized mode. The autonomous and sustained *SBT3.3* expression pattern should consequently be the basis to explain the memory-based characteristics of priming and IR, manifested only secondarily in hosts after a primary infection. Thus, SBT3.3 appears key in regulating this type of training effect leading to IR. Interestingly, MPK3 and MPK6 activation, and to a lesser extend also MPK4/MPK11, were enhanced in *SBT3.3OEX* plants following infection with *Ps*DC3000, and conversely, this activation was compromised in *sbt3.3* plants. These results are congruent with increased *OXI1* expression in *SBT3.3OEX* plants, a kinase required for MPK3 and MPK6 activation [33], and indicates that following *SBT3.3* expression, plants respond faster to pathogenic stimuli. Because the MPK3 and MPK6 activation is critical for priming [12], and activation requires OXI1 expression [33], our data suggest SBT3.3 positively modulates immune responses upstream of the MAPK kinase pathway, and confirms that accumulation of defense signaling components, such as SBT3.3 itself, prior to a secondary pathogen challenge is essential for priming and induced resistance.

Similarly, the observation that sole *SBT3.3* expression poises SA-responsive defense genes for enhanced activation following perception of a pathogenic cue provides further support to SBT3.3 as an integral component in mounting primed immunity. ChIP assays revealed this poising effect for enhanced gene expression triggered by SBT3.3 was mediated by selective increases of histone activation marks on the promoter region of SA-responsive genes. Since similar histone activation marks have been found to appear in SA–related genes when wild type plants are treated with the priming agent BTH [20], our data therefore strongly support a model where SBT3.3 positively mediates OXI1-mediated MPK activation, and concurrent chromatin remodeling at SA-responsive genes as specific hallmarks for primed immunity. Furthermore, the observation that immune priming, and similar chromatin remodeling of SA-responsive genes is mirrored in plants defective in the RdDM pathway [23] provides support for a hypothesis that the observed SBT3.3-mediated priming mechanism might be under similar epigenetic control. Moreover, the fact that *SBT3.3* gene expression is constitutively up in the *nrpd2* mutant favors this interpretation. Furthermore, (1) the observation that histone activation marks are established in the promoter of the *SBT3.3* gene and in the promoters of SA-dependent genes by individual SBT3.3 overexpression in transgenic plants; (2) the reproducibility of similar chromatin remodeling of the *SBT3.3* gene promoter in *nrpd2* plants; (3) its similar remodeling in Col-0 plants following BTH treatment; (4) and the observation that such chromatin remodeling is strongly abrogated in *sbt3.3* plants, further substantiates the importance of SBT3.3 gene activation as a prerequisite for establishment of immune priming.

How does then a proteolytic enzyme such as SBT3.3, which accumulates in the extracellular matrix trigger activation of such a complex signaling pathway mediating priming and IR? One simple explanation is via SBT3.3-mediated protein substrate processing that co-localizes extracellularly. This substrate could exist in a soluble form, or be an extracellular domain (ectodomain) of a larger protein, likely functioning as a receptor located in the plasma membrane. After proteolytic shedding of the ectodomain by SBT3.3, the receptor could become activated and initiate a down stream immune signaling process. This mechanism has been identified as common in activating a variety of signaling processes in animal through the involvement of protease-activated receptors (PARs), a group of receptors mediating different cellular processes including proinflammatory responses, and is also a common principle in various diseases [49]. Moreover, the proteolytic processing mechanism of an extracellular substrate is reminiscent of that mediating activation of innate immunity in invertebrates through the transmembrane Toll receptor, or Toll-like receptors (TLRs) in humans; PRR-type receptors consisting of a leucine-rich repeat (LRR) ectodomain, a transmembrane domain, and a cytosolic signaling domain [Toll/IL-1 receptor (TIR)], which becomes activated only after the binding of a proteolytically processed peptide ligand (*i.e.* spätzle) by complex cascades of CLIP-domain serine proteases [50] or less common following specific cleavage of the receptor ectodomain by extracellular proteases [51], [52]. Alternatively, SBT3.3 processes the extracellular substrate and the cleaved polypeptide can be released and function as a ligand recognized by a nearby specific extracellular receptor, which in turn can initiate a downstream signaling. Tornero et al., [30] reported SBT3.3 homologous P69C subtilase can specifically process LRP in disease tomato plants, an extracellular LRR-containing protein of unknown function, and the first subtilase substrate identified in plants. This suggesting SBT3.3 could similarly be involved in the cleavage and activation of LRR-containing proteins, including PRR-type receptors, which in turn may activate innate immune responses. The recent finding that the lectin receptor kinase (*Lec*RK)-VI.2, a member of the LRR-containing superfamily of RLKs proteins existing in Arabidopsis, is required for immune priming acting upstream of MPK-mediated signaling [53] can give further support to this hypothesis.

The results of the present study identified SBT3.3 as a determinant host factor mediating activation of primed immune responses. Our immediate future challenge is to identify the target substrate processed by this subtilase and elucidate the mechanism transducing the substrate into a signal for immune prime activation.

## Materials and Methods

### Plants growth conditions

*Arabidopsis thaliana* and *Nicotiana benthamiana* plants were grown in a growth chamber (19–23°C, 85% relative humidity, 100 mEm^−2^ sec^−1^ fluorescent illumination) on a 10-hr-light and 14-hr-dark cycle. All mutants are in Col-0 background; *nrpd2-2, npr1-1 and sid2-1* plants were previously described [23], [26], [54]. *sbt3.3-1*, *sbt3.3-2, sbt3.4-1 and sbt3.4-2* mutants and *SBT3.3OEX1* and *SBT3.3OEX2* overexpression lines were obtained from the Plant Subtilase Database Consortium (PSDB) (http://csbdb.mpimp-golm.mpg.de/csbdb/dbcawp/psdb.html).

### Gene constructs and transgenic lines

For the *SBT3.3-GFP* overexpressing construct, a full length cDNA for *SBT3.3* was amplified by PCR using *Pfu* DNA polymerase (Stratagene, San Diego, CA) and specific primers including Gateway adapters: BP SBT3.3 FW and BP SBT3.3 RV and recombined into pDONR207 using BP ClonaseMixII kit (Invitrogen). For the *SBT3.3m-GFP* construct, pDONR207+SBT3.3 vector was amplified using Phusion Hot Start II polymerase (Thermo Scientific) with SBT3.3m FW and SBT3.3m RV phosphorylated primers including a T^663^ to G^663^ mutation. The PCR product was then digested with DpnI restriction enzyme (Fermentas), purified by Zymoclean DNA Recovery Kit (Zymo Research) and religated using T4 Ligase (Fermentas). For *SBT3.3noSP-GFP* construct pDONR207+SBT3.3 vector was amplified with SBT3.3noSP FW and SBT3.3 RV primers and recombined into pDONR207 as described above. After sequencing, all constructs were recombined with pB7FWG destination vector using LR ClonaseMixII kit (Invitrogen) and introduced into *Arabidopsis* (Col-0) via *Agrobacterium* transformation. The *sid2-1 SBT3.3OEX* and *npr1-1 SBT3.3OEX* lines were generated by the genetic crossing of the *sid2-1* and *npr1-1* mutants, respectively, with a *35S:SBT3.3* transgenic line containing a single insertion of the transgen. The *sbt3.3 SBT3.3-GFP* and *sbt3.3 SBT3.3m-GFP* lines were generated by direct genetic transformation of *sbt3.3-1* plants with *SBT3.3-GFP* and *SBT3.3m-GFP* gene constructs, respectively. The selected lines were those expressing higher levels of the corresponding transgene as determined by RT-PCR of RNA preparations. For PCR-based detection of the *sid2-1* mutant allele the primers used were *sid2-1 Fw* and *sid2-1 Rv* GCA GTC CGA AAG ACG ACC TCG AG and CTA TCG AAT GAT TCT AGA AGA AGC), followed by *Mun* I digestion of the ensuing fragment (the mutant allele *sid2*-*1* cannot be digested). For PCR-based detection of the *npr1-1* mutant allele, the primers used were *npr1-1 Fw* and *npr1-1 Rv* (5′-ATGTCTCGAATGTACATAAGGC-3′ and 5′-CTCAGTTTCCTAATAGAGAGG-3′).

### Transient expression in *Nicotiana benthamiana* leaves

Almost fully expanded leaves were infiltrated with a suspension of *Agrobacterium tumefaciens* C58 bearing the relevant construct in 10 mM MES pH 5.6, 10 mM MgCl_2_, 150 µM acetosyringone at an OD_600_ = 0.5. After 3 days, fluorescence was analyzed in infiltrated leaves by confocal microscopy. For co-infiltration, *Agrobacterium* cultures grown separately and processed as indicated above, were adjusted to an O.D. = 0.5, and mixed prior to infiltration. Agrobacterium expressing the viral silencing suppressor P19 was included in all infiltrations.

### Fluorescence microscopy

GFP/YFP fluorescence in inoculated plants was monitored using Nikon SMZ800, and Leica MZ16F microscopes.

### Gene expression analysis

Total RNA was extracted using TRIzol reagent (Invitrogen) following the manufacturer's recommendations and further purified by lithium chloride precipitation. For reverse transcription, the RevertAid H Minus First Strand cDNA Synthesis Kit (Fermentas Life Sciences) was used. Quantitative PCR (qPCR) amplifications and measurements were performed using an ABI PRISM 7000 sequence detection system, and SYBR-Green (Perkin-Elmer Applied Biosystems). *ACTIN2* was chosen as the reference gene. The primers used to amplify the different genes and DNA regions, and the PCR conditions employed for genotyping T-DNA insertions, and RT-PCR and qRT-PCR experiments are provided in the supporting information file Text S1. RT-qPCR analyses were performed at least three times using sets of cDNA samples from independent experiments.

### Microarray hybridization and data analysis

Affymetrix microarrays (*Arabidopsis* ATH1 genome array) containing 22,810 probe sets were used. Labeling and hybridization on the ATH1 microarrays were performed according to the manufacturer's instructions (www.affymetrix.com/support/technical/manual/expression_manual.affx). Global analysis of gene expression was performed by using Affymetrix MAS5.0. SAM analysis (Significance Analysis of Microarrays software package) was conducted for *A. thaliana* triplicate samples between *csb3* plants and control plants using a *q* value≤0.05 and a fold change cutoff ≥2 to identify the genes differentially expressed in the mutant. We searched GO enrichment information for the differently expressed probe sets using EasyGO (http://bioinformatics.cau.edu.cn/easygo/ category_treeBrowse.html). We applied *χ* ^2^ analysis for the biological process search, and the cutoff for false discovery rate (FDR) was adjusted using a *p* value of 0.0001. GeneChip data set are available in a MIAME-compliant format through GEO (accession no. GSE35507).

### Bacterial and oomycete bioassays

Bacterial strains were grown overnight and used to infect 5-week-old *Arabidopsis* leaves by infiltration and bacterial growth determined following [23], [54]. Twelve samples were used for each data point and represented as the mean ± SEM of log c.f.u./cm^2^. *H. arabidopsidis* WACO9 sporangia were obtained by washing sporulating Col-0 leaves in 10 mM MgSO_4_, collected by centrifugation, and resuspended in 10 mM MgSO_4_ to a final density of 5×10^4^ sporangia per mL as described [25]. Three-week-old seedlings were challenge inoculated with *H. arabidopsidis* by spraying with 10 mM MgSO_4_ containing 5×10^4^ conidiospores per mL. Inoculated plants were maintained at 17°C and 100% relative humidity. Disease symptoms were scored for about 200 leaves per treatment at 7 days after challenge. For determining leaf colonization, infected leaves were stained with lactophenol trypan-blue and examined microscopically at 7 days after inoculation, as described [25] and scored on each leaf in the following classes: I, no colonization; II, low tissue colonization (<25% of leaf area colonized); III, medium tissue colonization (25–50% of leaf area colonized); IV, high tissue colonization (>50% of leaf area colonized). Sporulation was expressed as intensity of pathogen sporulation on each leaf: I, no sporulation; II, <50% of the leaf area covered by sporangiophores; III, >50% of the leaf area covered by sporangiophores; and IV, heavily covered with sporangiophores, with additional chlorosis and leaf collapse. When indicated, oomycete spore counting was performed as previously described [26].

### Chromatin immunoprecipitation

Chromatin isolation and immunoprecipitation were performed as described [55]. Chip samples, derived from three biological replicates, were amplified in triplicate and measured by quantitative PCR using primers for *PR-1*, *WRKY6*, *WRKY53* and *Actin2* as reported [21]. The rest of primers are described in Text S1 file. All ChIP experiments were performed in three independent biological replicates. The antibodies used for immunoprecipitation of modified histones from 2 g of leaf material were antiH3K4m3 (#07-473 Millipore) and antiH3K4ac (#07-352 Millipore).

### Western blot

Protein crude extracts were prepared by homogenizing ground frozen leaf material with Tris-buffered saline (TBS) supplemented with 5 mM DTT, protease inhibitor cocktail (Sigma-Aldrich), and protein phosphatase inhibitors (PhosStop, Roche). Protein concentration was measured using Bradford reagent; 25 µg of total protein was separated by SDS-PAGE (12% acrylamide w/v) and transferred to nitrocellulose filters. The filter was stained with Ponceau-S after transfer, and used as a loading control.

## Supporting Information

Figure S1 **Pie chart categorizing genes which are differentially expressed in Col-0 and** ***csb3*** **plants.** Genes with p-values less than 0,05 and fold changes greater than 2 are included. These genes are grouped based on their functional annotations and normed to frequency of class over the genome using Classification Superviewer (www.bar.utoronto.ca). Number of genes of each class is indicated.
(TIF)

Figure S2 **Bootstrapped consensus neighbour-joining tree generated from an alignment of the annotated 56 AtSBT full-length protein sequences.** Gene expression analysis of the 56 *Arabidopsis* subtilase members in response to SA, MeJA, ACC, ABA, *P. syringae* DC3000 (Ps), *B. cinerea* (Bc) and oxydative stress (OX). Response analyzed by microarray database analysis using the Botany Array Resource program (Toufighi et al., 2005). AtSBT3.3 (At1g32960) is highlighted in bold.
(TIF)

Figure S3 **Deduced amino acid sequence of the gene encoding SBT3.3 subtilase.** The catalytically important Asp, His, Asn, and Ser residues are in boldface typed in blue and indicated with asterisks. The propeptide domain in indicated in green. The signal peptide is indicated in red. Potential consensus sequences for N-glycosylation are marked in orange.
(TIF)

Figure S4 **Comparative induction of the SA-dependent** ***PR-1 gene*** **and the** ***SBT3.3*** **gene expression by application of 1 µM Fgl22.** RT-qPCR analysis showing gene expression in mock- (white columns) and Fgl22-treated (solid columns) Col-0 seedlings 1 h after treatment. Data represent the mean ± SD; n = 3 biological replicates. Expression was normalized to the expression of the constitutive *ACT2* gene and then to the expression in time 0 Col-0 plants.
(TIF)

Figure S5 **Disease responses to** ***H. arabidopsidis*** **as assessed by direct counting of spore production on inoculated plants.** To quantify resistance to *H. arabidopsidis*, production of spores was counted 7 days after inoculation. Plants carrying the *sbt3.3* mutations were highly resistant to this pathogen while overexpression of SBT3.3 conferred enhanced resistance to this pathogen. Error bars represent standard deviation (n = 30). Asterisks indicate statistical differences to Col-0 (P<0.05) using Student's *t* test.
(TIF)

Figure S6 **Extracellular localization of SBT3.3-GFP in transgenic Arabidopsis leaves by confocal microscopy.** Expression of SBT3.3-GFP in transgenic Arabidopsis results in a uniform extracellular fluorescence. Upper panel shows GFP localization in leaves of transgenic plants expressing SBT3.3-GFP. Lower panel shows a magnification of the tissue section shown in the upper panel.
(TIF)

Figure S7 **Expression of a missense mutant of SBT3.3 (S555A; SBT3.3m) in** ***N. benthamiana*** **leaves no longer promotes accumulation of the endogenous P69 subtilase or PR-1a proteins.** Total protein extracts from *N. benthamiana leaves* transitorily overexpressing GFP alone, SBT3.3-GFP or SBT3.3m-GFP fusion proteins were separated on a 10% SDS-PAGE gel, transferred to nitrocellulose and the blots revealed with anti-GFP antibodies (α-GFP; upper panels), anti-P69 antibodies (α-P69) and anti-PR-1a antibodies (α-PR-1a). Total protein extracts from empty *A. tumefaciens* agroinfiltrated *N. benthamiana* leaves (mock) were used as controls. The sizes of the marker proteins are indicated by arrows. Equal protein loading was monitored by staining the filters with Ponceau.
(TIF)

Figure S8 **Transgenic** ***35S::SBT3.3-GFP*** **plants, but not transgenic** ***35S::SBT3.3m-GFP*** **plants, show enhanced disease resistance towards** ***Ps*** **DC3000.** Col-0 plants were genetically transformed with *35S::SBT3.3-GFP* and *35S::SBT3.3m-GFP* and stable homozygous lines sowing expression of the transgene were selected for evaluation of the resistance phenotype towards *Ps*DC3000 in comparison to Col-0 plants and *SBT3.3OEX1* plants. Five-week-old plants of the indicated genetic backgrounds were inoculated with *Ps*DC3000 and the bacterial growth measured at five days post-inoculation. Error bars represent standard deviation (n = 12). Asterisks indicate statistical differences to Col-0 (P<0.05) using Student's *t* test.
(TIF)

Figure S9 **Transgenic** ***sbt3.3*** **plants expressing SBT3.3-GFP lose the enhanced disease susceptibility to** ***P. syringae*** **DC3000.** *sbt3.3* and Col-0 plants were stably transformed with a *35S::SBT3.3-GFP* construct and two independent stable homozygous lines sowing expression of the transgene were selected for evaluation of the resistance phenotype towards *Ps*DC3000 in comparison to untransformed plants. Five-week-old plants of the indicated genetic backgrounds were inoculated with *Ps*DC3000 and the bacterial growth measured at five days post-inoculation. Error bars represent standard deviation (n = 12). Asterisks indicate statistical differences to Col-0 (P<0.05) using Student's *t* test.
(TIF)

Table S1 **Genes up and down regulated in the csb3 mutant.**
(XLSX)

Table S2 **Defense-related genes up-regulated (≥2 fold) in the Arabidopsis** ***csb3*** **mutant with respect to wild type (wt) plants.** AtSBT3.3 (At1g32960) is highlighted in bold.
(TIF)

Text S1 **Primer sequences.**
(XLSX)
