# Correction: An Extracellular Subtilase Switch for Immune Priming in Arabidopsis

**DOI:** 10.1371/journal.ppat.1006003

**Published:** 2016-11-02

**Authors:** Vicente Ramírez, Ana López, Brigitte Mauch-Mani, Ma José Gil, Pablo Vera

The authors would like to correct Fig 2. The figure contains two errors. In Fig 2A, the sbt3.4–2 representative picture was duplicated from Figure 5A (NPR1-H). These errors occurred during assembly of the final figure. Additionally, in Fig 2C, the middle Ponceau panel (ssbt3.3–1 experiment) was duplicated in the bottom Ponceau panel (sbt3.3–2 experiment). The authors have provided a corrected Fig 2 here. The authors confirm that these changes do not alter their findings. The authors have provided raw, uncropped blots as Supporting Information.

**Fig 2:**
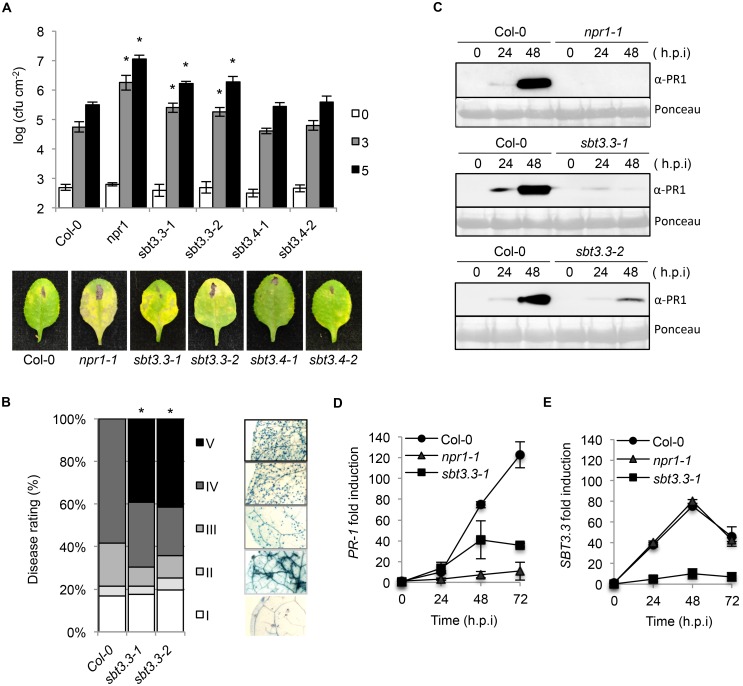
SBT3.3 loss of function increases disease susceptibility to P. syringae DC3000 and H. arabidopsidis. (A) Five-week-old plants were inoculated with PsDC3000. Zero (white bars), three (grey bars) and five (black bars) days after inoculation, the bacterial growth was measured. Error bars represent standard deviation (n = 12). Asterisks indicate statistical differences to Col-0 (P<0.05) using Student's t test. Below are representatives of inoculated leaves of the indicated genotypes. (B) Quantification of H. arabidopsidis conidia development on Col-0, and sbt3.3–1 and sbt3.3–2 mutants. Asterisks indicate statistically different distributions of disease severity classes compared with Col-0 plants (χ2 test; α  = 0.05). (C) Western blots with anti-PR1 antibodies reveals inhibition of PR1 induced accumulation in nrp1, sbt3.3–1 and sbt3.3–2 mutant plants, compared to Col-0, following inoculation with PsDC3000. The experiments were repeated three times with similar results. (D–E) Time-course RT-qPCR analysis showing PR-1 (D) and SBT3.3 (E) gene expression in Col-0, sbt3.3–1, and npr1-1 plants after infection with PsDC3000. Data represent the mean ± SD; n = 3 replicates and gene expression given as in Fig 1.

## Supporting Information

S1 File Raw, uncropped blots. (PDF)
