# Supplementary figures and images for: An Extracellular Subtilase Switch for Immune Priming in Arabidopsis
Source: PLoS Pathog. 2013 Jun 20;9(6):e1003445. doi: 10.1371/journal.ppat.1003445 (PMC3688555; doi:10.1371/journal.ppat.1003445)

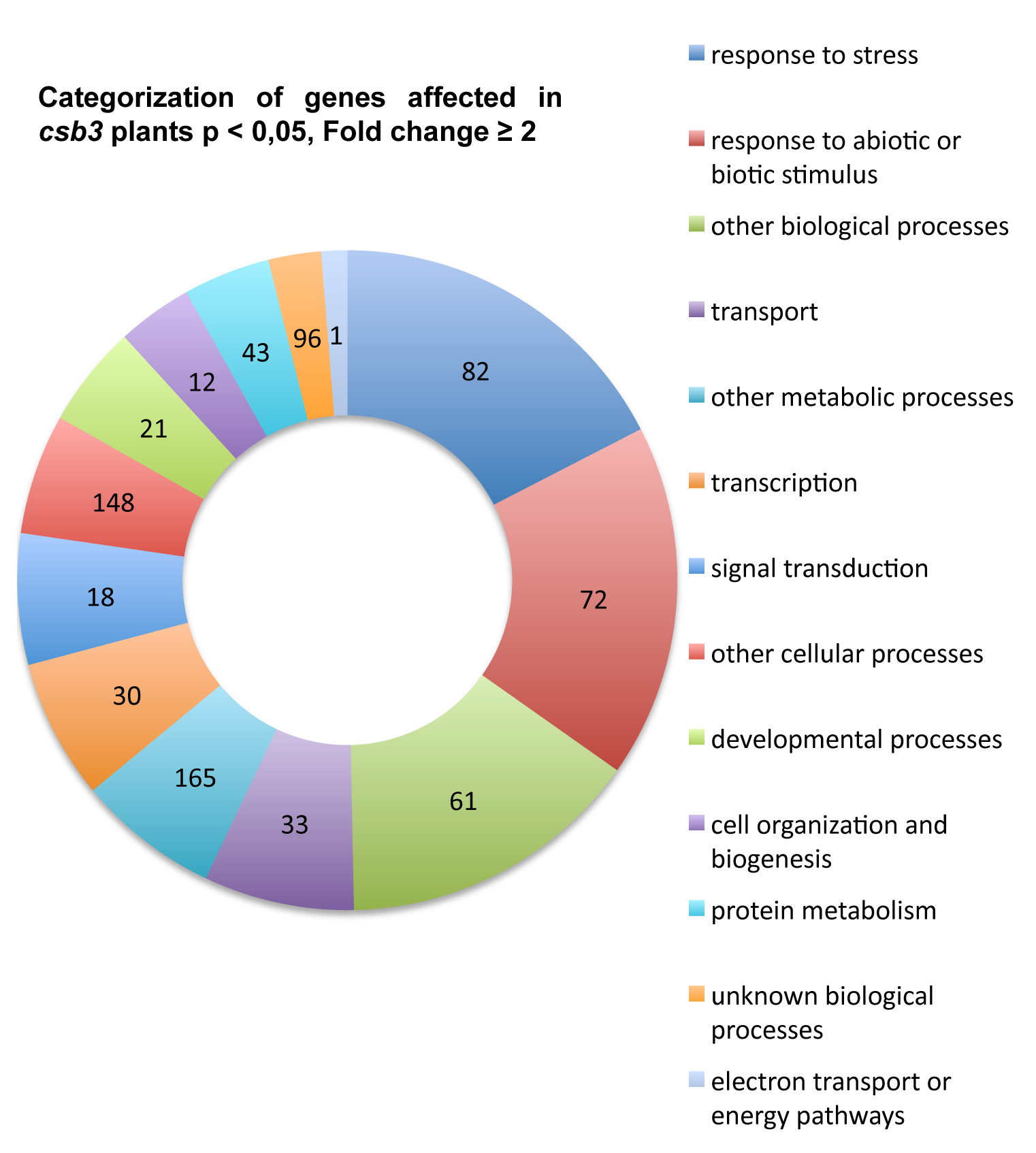

Supplement: Figure S1 — Pie chart categorizing genes which are differentially expressed in Col-0 and csb3 plants. Genes with p-values less than 0,05 and fold changes greater than 2 are included. These genes are grouped based on their functional annotations and normed to frequency of class over the genome using Classification Superviewer (www.bar.utoronto.ca). Number of genes of each class is indicated. (TIF) [file ppat.1003445.s001.tif]

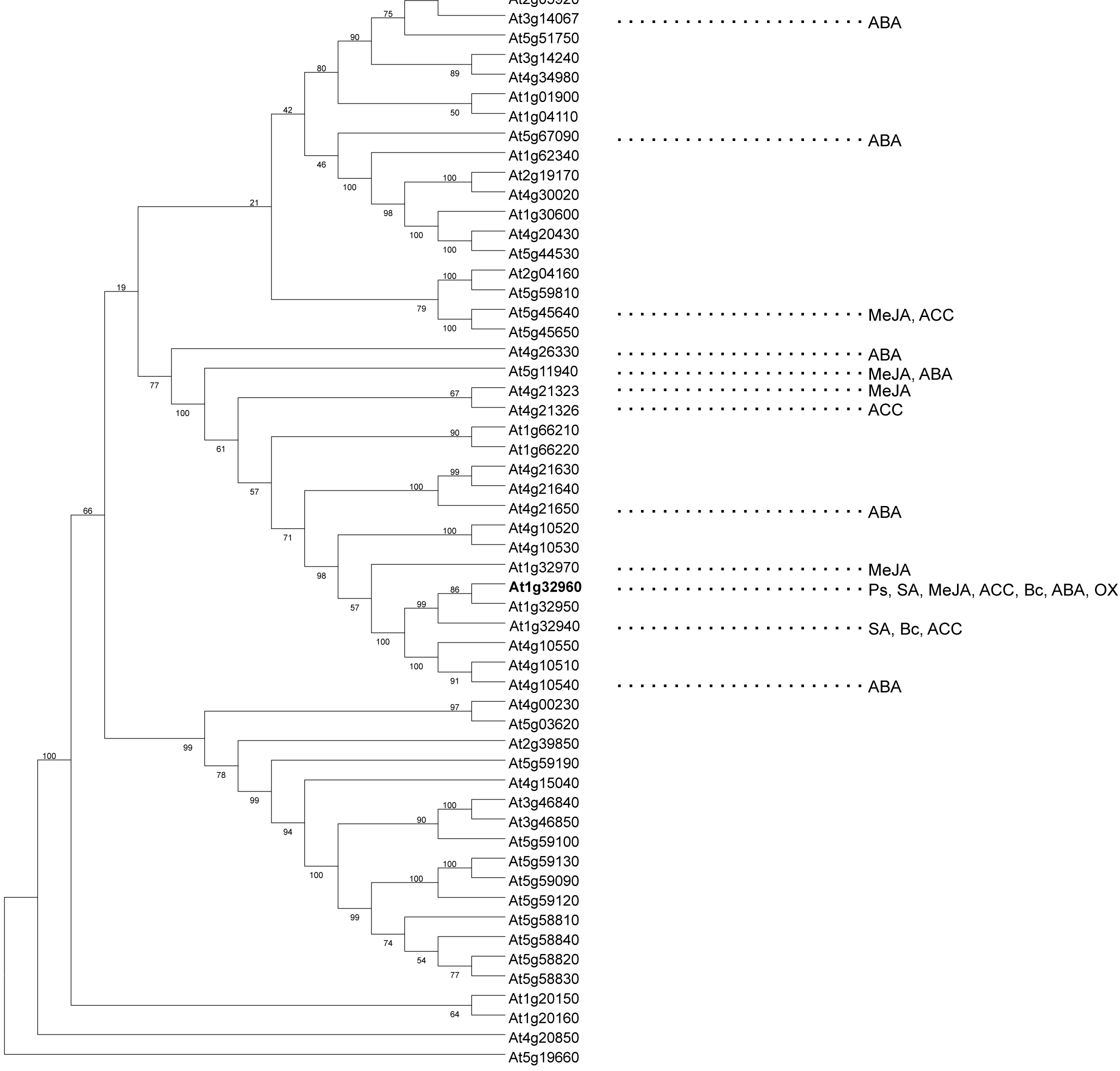

Supplement: Figure S2 — Bootstrapped consensus neighbour-joining tree generated from an alignment of the annotated 56 AtSBT full-length protein sequences. Gene expression analysis of the 56 Arabidopsis subtilase members in response to SA, MeJA, ACC, ABA, P. syringae DC3000 (Ps), B. cinerea (Bc) and oxydative stress (OX). Response analyzed by microarray database analysis using the Botany Array Resource program (Toufighi et al., 2005). AtSBT3.3 (At1g32960) is highlighted in bold. (TIF) [file ppat.1003445.s002.tif]

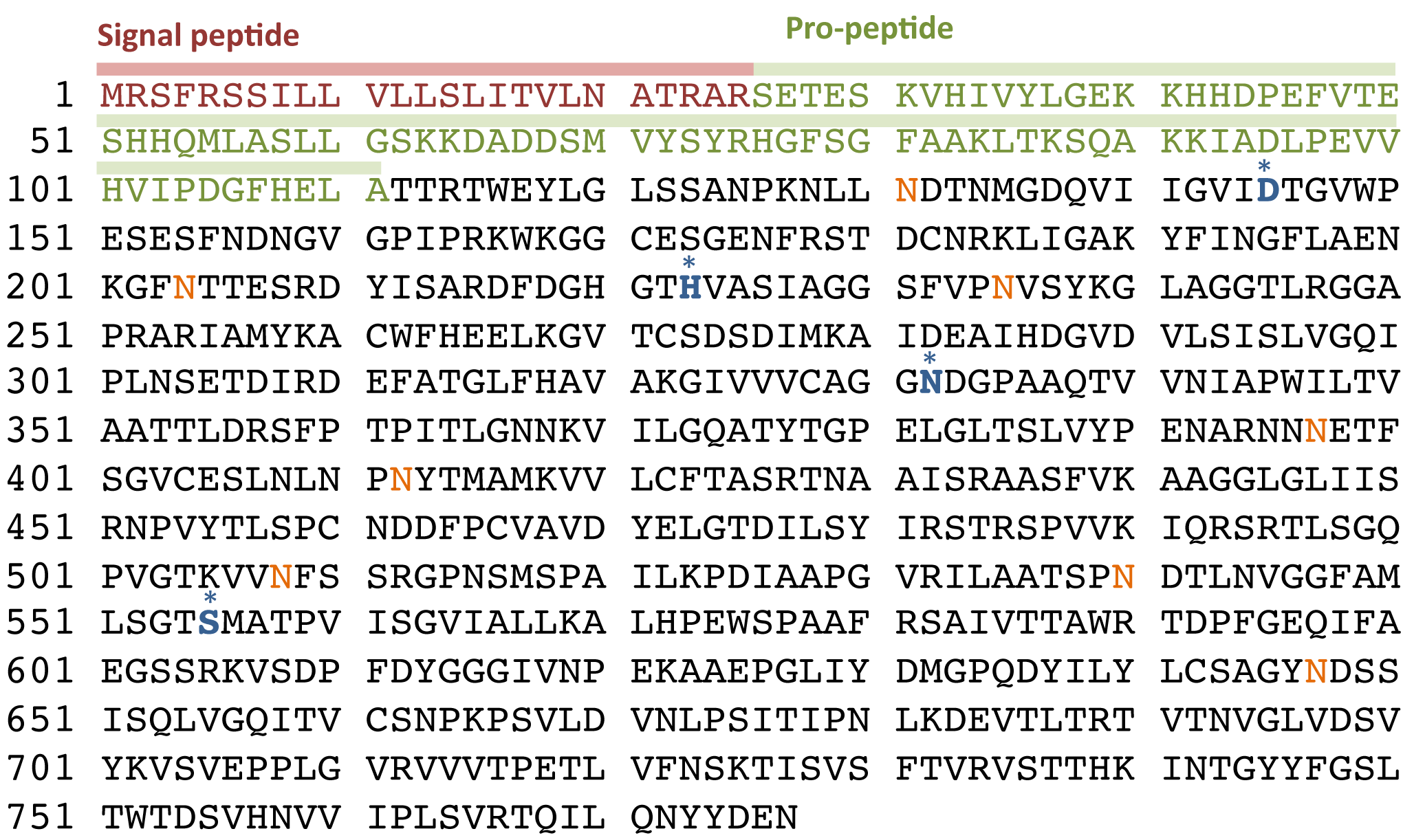

Supplement: Figure S3 — Deduced amino acid sequence of the gene encoding SBT3.3 subtilase. The catalytically important Asp, His, Asn, and Ser residues are in boldface typed in blue and indicated with asterisks. The propeptide domain in indicated in green. The signal peptide is indicated in red. Potential consensus sequences for N-glycosylation are marked in orange. (TIF) [file ppat.1003445.s003.tif]

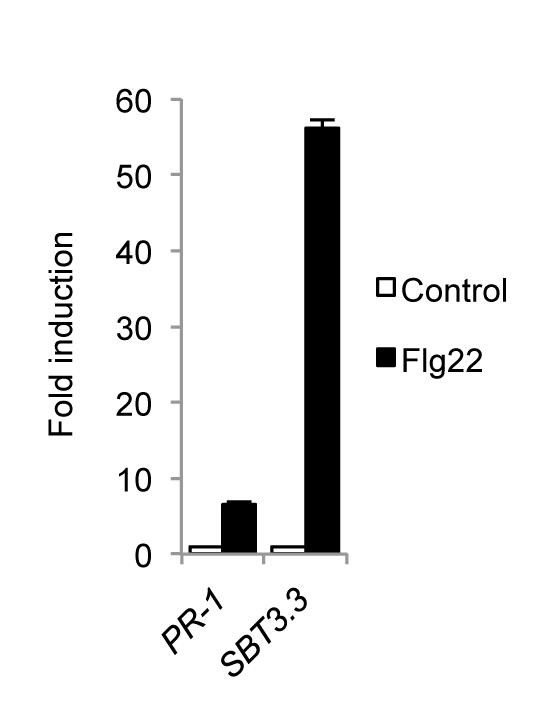

Supplement: Figure S4 — Comparative induction of the SA-dependent PR-1 gene and the SBT3.3 gene expression by application of 1 µM Fgl22. RT-qPCR analysis showing gene expression in mock- (white columns) and Fgl22-treated (solid columns) Col-0 seedlings 1 h after treatment. Data represent the mean ± SD; n = 3 biological replicates. Expression was normalized to the expression of the constitutive ACT2 gene and then to the expression in time 0 Col-0 plants. (TIF) [file ppat.1003445.s004.tif]

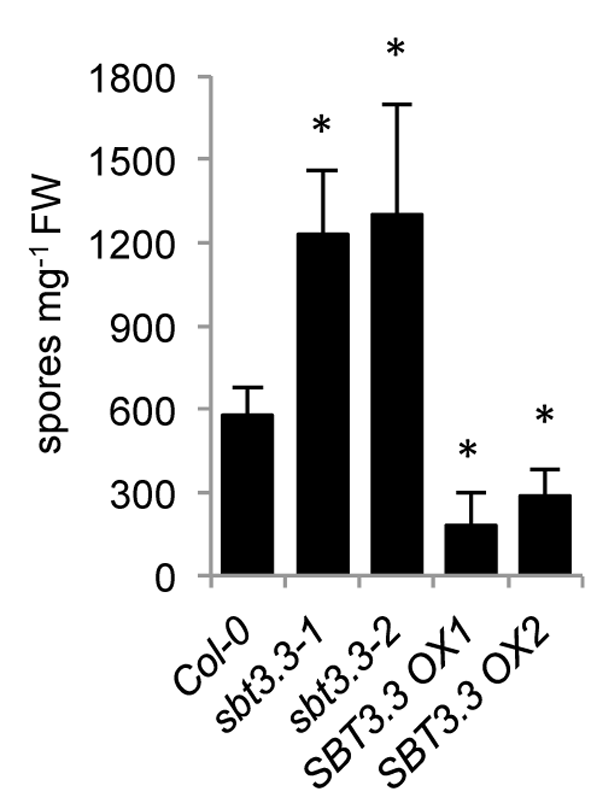

Supplement: Figure S5 — Disease responses to H. arabidopsidis as assessed by direct counting of spore production on inoculated plants. To quantify resistance to H. arabidopsidis, production of spores was counted 7 days after inoculation. Plants carrying the sbt3.3 mutations were highly resistant to this pathogen while overexpression of SBT3.3 conferred enhanced resistance to this pathogen. Error bars represent standard deviation (n = 30). Asterisks indicate statistical differences to Col-0 (P<0.05) using Student's t test. (TIF) [file ppat.1003445.s005.tif]

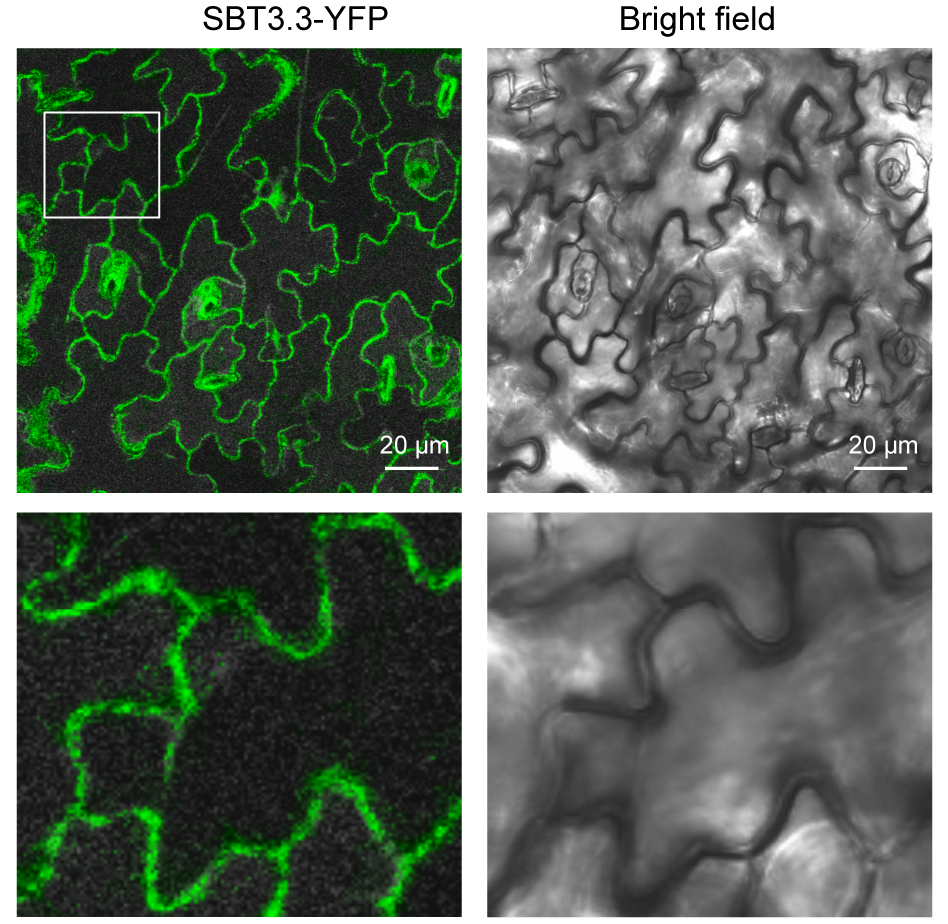

Supplement: Figure S6 — Extracellular localization of SBT3.3-GFP in transgenic Arabidopsis leaves by confocal microscopy. Expression of SBT3.3-GFP in transgenic Arabidopsis results in a uniform extracellular fluorescence. Upper panel shows GFP localization in leaves of transgenic plants expressing SBT3.3-GFP. Lower panel shows a magnification of the tissue section shown in the upper panel. (TIF) [file ppat.1003445.s006.tif]

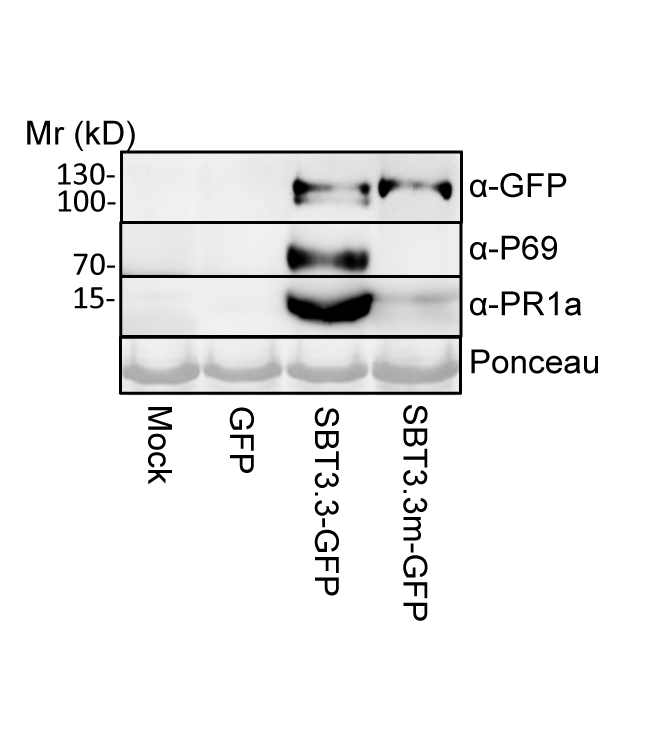

Supplement: Figure S7 — Expression of a missense mutant of SBT3.3 (S555A; SBT3.3m) in N. benthamiana leaves no longer promotes accumulation of the endogenous P69 subtilase or PR-1a proteins. Total protein extracts from N. benthamiana leaves transitorily overexpressing GFP alone, SBT3.3-GFP or SBT3.3m-GFP fusion proteins were separated on a 10% SDS-PAGE gel, transferred to nitrocellulose and the blots revealed with anti-GFP antibodies (α-GFP; upper panels), anti-P69 antibodies (α-P69) and anti-PR-1a antibodies (α-PR-1a). Total protein extracts from empty A. tumefaciens agroinfiltrated N. benthamiana leaves (mock) were used as controls. The sizes of the marker proteins are indicated by arrows. Equal protein loading was monitored by staining the filters with Ponceau. (TIF) [file ppat.1003445.s007.tif]

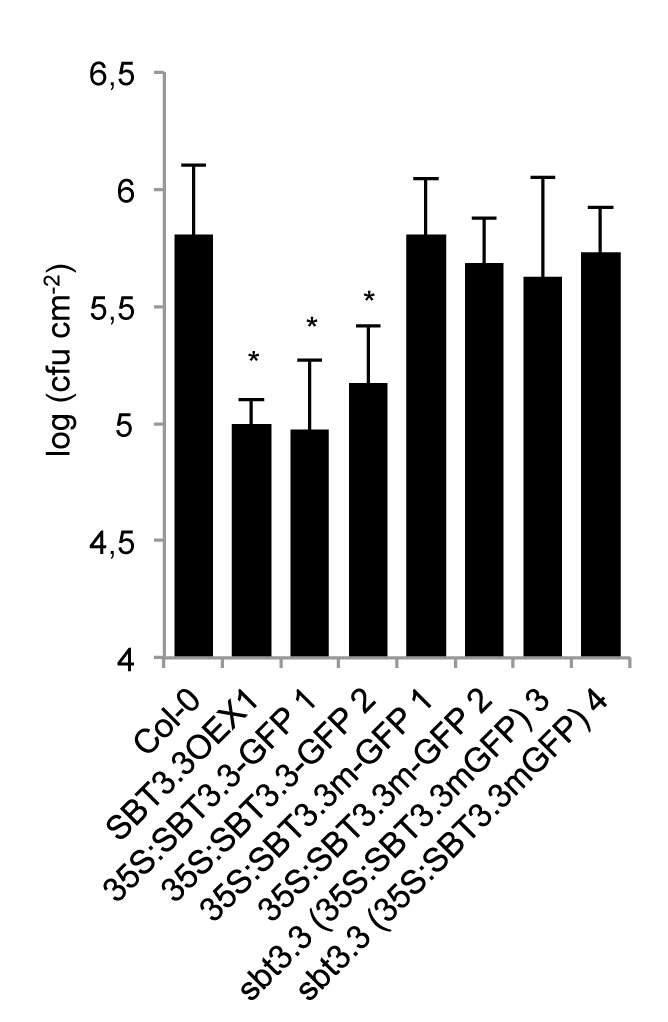

Supplement: Figure S8 — Transgenic 35S::SBT3.3-GFP plants, but not transgenic 35S::SBT3.3m-GFP plants, show enhanced disease resistance towards Ps DC3000. Col-0 plants were genetically transformed with 35S::SBT3.3-GFP and 35S::SBT3.3m-GFP and stable homozygous lines sowing expression of the transgene were selected for evaluation of the resistance phenotype towards PsDC3000 in comparison to Col-0 plants and SBT3.3OEX1 plants. Five-week-old plants of the indicated genetic backgrounds were inoculated with PsDC3000 and the bacterial growth measured at five days post-inoculation. Error bars represent standard deviation (n = 12). Asterisks indicate statistical differences to Col-0 (P<0.05) using Student's t test. (TIF) [file ppat.1003445.s008.tif]

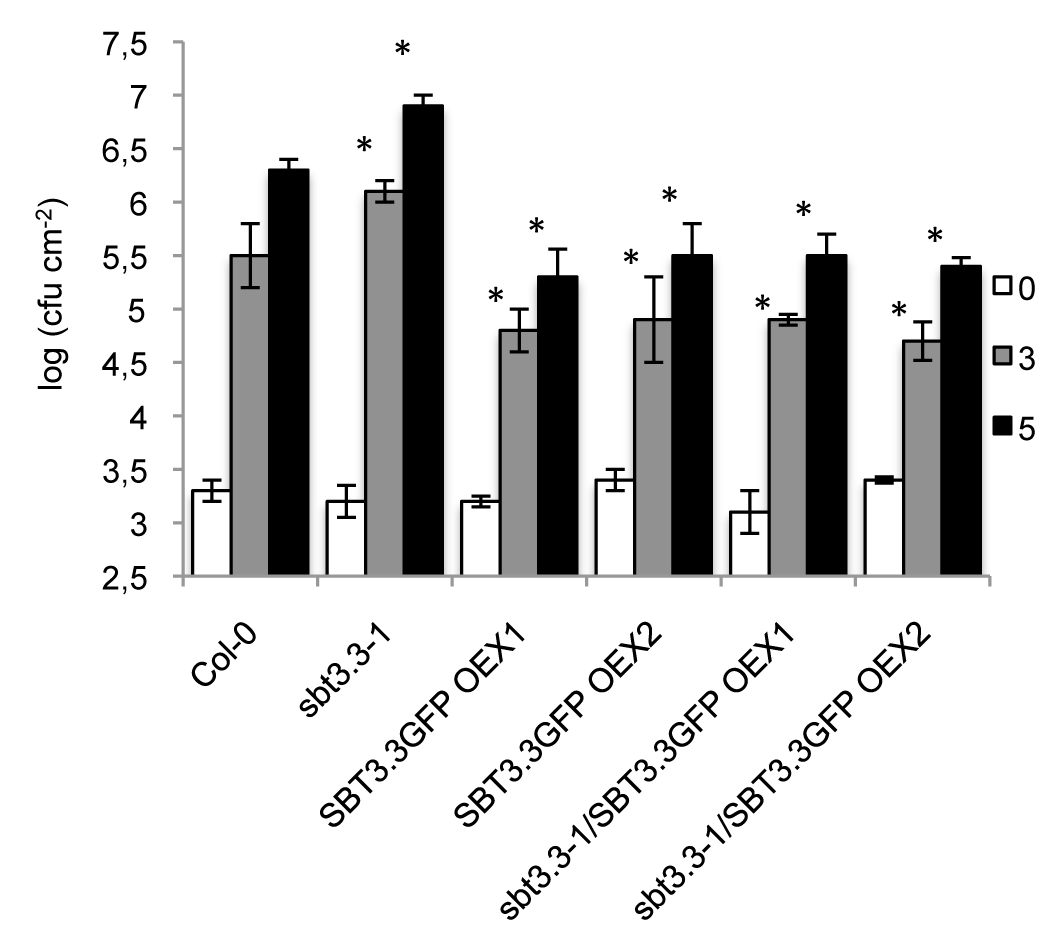

Supplement: Figure S9 — Transgenic sbt3.3 plants expressing SBT3.3-GFP lose the enhanced disease susceptibility to P. syringae DC3000. sbt3.3 and Col-0 plants were stably transformed with a 35S::SBT3.3-GFP construct and two independent stable homozygous lines sowing expression of the transgene were selected for evaluation of the resistance phenotype towards PsDC3000 in comparison to untransformed plants. Five-week-old plants of the indicated genetic backgrounds were inoculated with PsDC3000 and the bacterial growth measured at five days post-inoculation. Error bars represent standard deviation (n = 12). Asterisks indicate statistical differences to Col-0 (P<0.05) using Student's t test. (TIF) [file ppat.1003445.s009.tif]

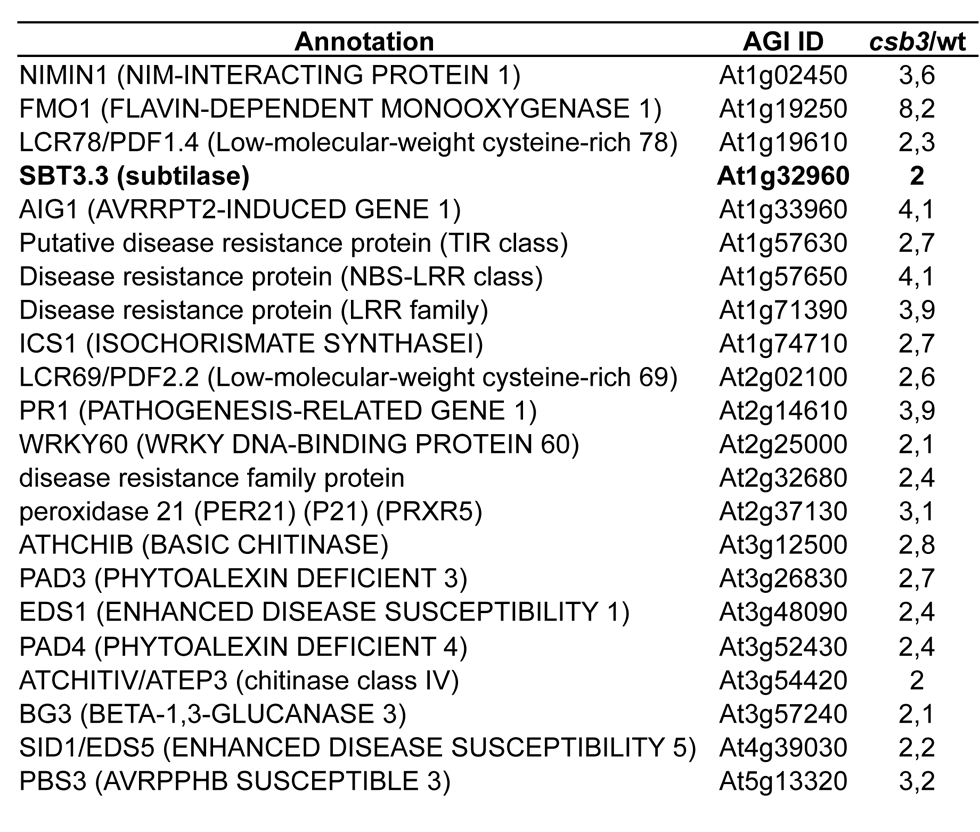

Supplement: Table S2 — Defense-related genes up-regulated (≥2 fold) in the Arabidopsis csb3 mutant with respect to wild type (wt) plants. AtSBT3.3 (At1g32960) is highlighted in bold. (TIF) [file ppat.1003445.s011.tif]
