# Supplementary figures and images for: Correction: An Extracellular Subtilase Switch for Immune Priming in Arabidopsis
Source: PLoS Pathog. 2016 Nov 2;12(11):e1006003. doi: 10.1371/journal.ppat.1006003 (PMC5091755; doi:10.1371/journal.ppat.1006003)

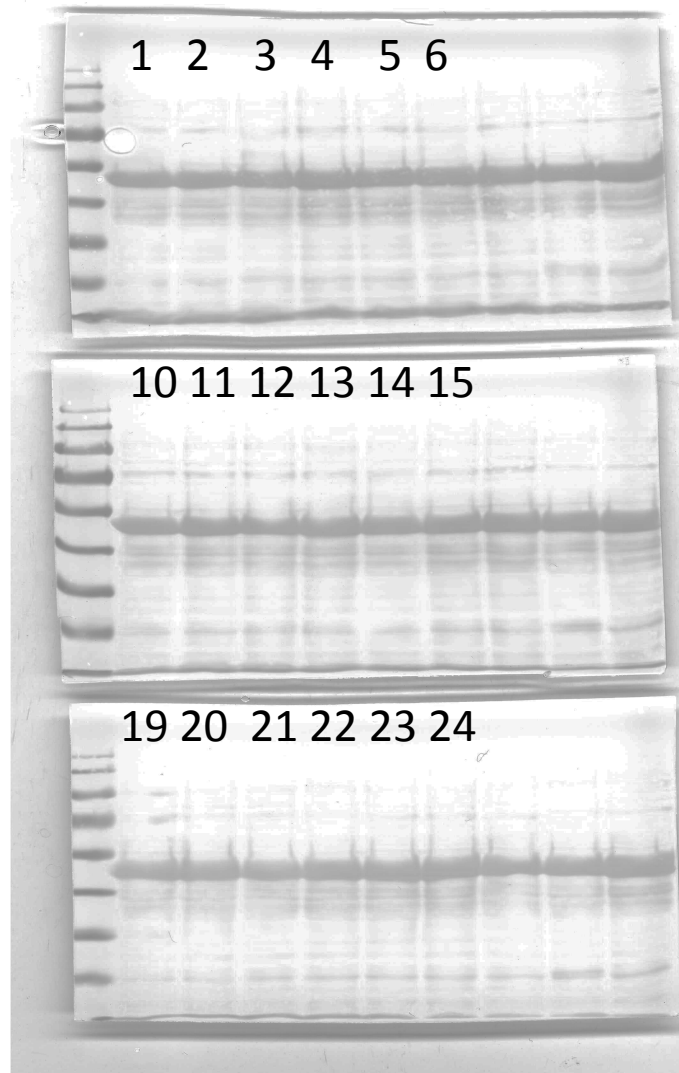

1-Col0 T0  
2-Col0 24  
3-Col0 48  
4-npr1 T0  
5-npr1 T24  
6-npr1T48

10-Col0 T0  
11-Col0 24  
12-Col0 48  
13-sbt3.3 T0  
14-sbt3.3 T24  
15-sbt3.3 T48

19-Col0 T0  
20-Col0 24  
21-Col0 48  
22-sbt3.3-2 T0  
23-sbt3.3-2 T24  
24-sbt3.3-2 T48

Supplement: S1 File — (PDF) [file ppat.1006003.s001.pdf]
